# The free fatty acid–binding pocket is a conserved hallmark in pathogenic β-coronavirus spike proteins from SARS-CoV to Omicron

**DOI:** 10.1126/sciadv.adc9179

**Published:** 2022-11-23

**Authors:** Christine Toelzer, Kapil Gupta, Sathish K. N. Yadav, Lorna Hodgson, Maia Kavanagh Williamson, Dora Buzas, Ufuk Borucu, Kyle Powers, Richard Stenner, Kate Vasileiou, Frederic Garzoni, Daniel Fitzgerald, Christine Payré, Gunjan Gautam, Gérard Lambeau, Andrew D. Davidson, Paul Verkade, Martin Frank, Imre Berger, Christiane Schaffitzel

**Affiliations:** ^1^ School of Biochemistry, University of Bristol, 1 Tankard’s Close, Bristol BS8 1TD, UK.; ^2^ Bristol Synthetic Biology Centre BrisSynBio, 24 Tyndall Ave, Bristol BS8 1TQ, UK.; ^3^ Imophoron Ltd., St. Philips Central, Albert Rd, Bristol BS2 0XJ, UK.; ^4^ Cellular and Molecular Medicine, University of Bristol, University Walk, Bristol BS8 1TD, UK.; ^5^ Max Planck Bristol Centre for Minimal Biology, Cantock’s Close, Bristol BS8 1TS, UK.; ^6^ Halo Therapeutics Ltd., St. Philips Central, Albert Rd, Bristol BS2 0XJ, UK.; ^7^ Université Côte d’Azur, Centre National de la Recherche Scientifique, Institut de Pharmacologie Moléculaire et Cellulaire, Valbonne Sophia Antipolis, France.; ^8^ Biognos AB, Box 8963, 40274 Göteborg, Sweden.; ^9^ School of Chemistry, University of Bristol, Cantock’s Close, Bristol BS8 1TS, UK.

## Abstract

As coronavirus disease 2019 (COVID-19) persists, severe acute respiratory syndrome coronavirus 2 (SARS-CoV-2) variants of concern (VOCs) emerge, accumulating spike (S) glycoprotein mutations. S receptor binding domain (RBD) comprises a free fatty acid (FFA)–binding pocket. FFA binding stabilizes a locked S conformation, interfering with virus infectivity. We provide evidence that the pocket is conserved in pathogenic β-coronaviruses (β-CoVs) infecting humans. SARS-CoV, MERS-CoV, SARS-CoV-2, and VOCs bind the essential FFA linoleic acid (LA), while binding is abolished by one mutation in common cold–causing HCoV-HKU1. In the SARS-CoV S structure, LA stabilizes the locked conformation, while the open, infectious conformation is devoid of LA. Electron tomography of SARS-CoV-2–infected cells reveals that LA treatment inhibits viral replication, resulting in fewer deformed virions. Our results establish FFA binding as a hallmark of pathogenic β-CoV infection and replication, setting the stage for FFA-based antiviral strategies to overcome COVID-19.

## Teaser

The free-fatty acid-binding pocket is fully conserved in severely pathogenic human β-coronaviruses.

## INTRODUCTION

Severe acute respiratory syndrome coronavirus 2 (SARS-CoV-2) causes the ongoing coronavirus disease 2019 (COVID-19) pandemic with millions of lives lost, damaging communities and economies. Human coronaviruses were previously only known to cause mild diseases of the upper respiratory tract until the emergence of the pathogenic coronaviruses SARS-CoV and Middle East respiratory syndrome coronavirus (MERS-CoV) in 2002 and 2012, respectively. Both cause severe pneumonias with a high incidence of mortality. Pathogenic SARS-CoV-2, SARS-CoV, MERS-CoV, and the endemic common cold–causing HCoV-OC43 and HCoV-HKU1 viruses all belong to the β-coronavirus (β-CoV) genus of the family Coronaviridae. During the present pandemic, numerous variants of concern (VOCs) have emerged, exhibiting increased transmissibility, increased risk of reinfection, and reduced vaccine efficiency (*1*), highlighting the urgent need for effective antiviral treatment strategies. These VOCs include the SARS-CoV-2 lineages B.1.1.7 (Alpha), B.1.351 (Beta), P.1 (Gamma), B.1.617.2 (Delta), and, most recently, B.1.1.529 (Omicron) (*2*).

The trimeric spike (S) glycoprotein decorates the surface of coronaviruses and mediates entry into host cells. S is the major antigen recognized by neutralizing antibodies and the main target for vaccine development (*3*). SARS-CoV S and SARS-CoV-2 S both bind to human angiotensin-converting enzyme 2 (ACE2) receptor on the host cell surface (*4*–*6*), MERS-CoV S binds to dipeptidyl-peptidase-4 (DPP4) (*5*, *7*), while the HCoV-HKU1 and HCoV-OC43 S proteins bind to the *N*-acetyl-9-*O*-acetylneuraminic acid receptor (*8*). SARS-CoV-2 S is cleaved by host cell proteases into the receptor binding fragment S1 and the partially buried fusion fragment S2 (*4*). S1 is composed of the N-terminal domain (NTD), the receptor binding domain (RBD) with a receptor binding motif (RBM), and two C-terminal domains (CTDs). S2 mediates fusion of the viral envelope with host cell membranes and is composed of the fusion peptide, heptad repeats, transmembrane domain, and cytoplasmic C terminus (*9*). In the prefusion conformation, the RBDs in the S trimer can alternate between closed (“down”) and open (“up”) conformations. SARS-CoV and SARS-CoV-2 S require RBD up conformations for interaction with ACE2 (*6*, *9*, *10*) for cell entry.

In our previous SARS-CoV-2 S structure, we discovered a free fatty acid (FFA) bound to a hydrophobic pocket in the RBD (*11*). Mass spectroscopy identified this ligand as linoleic acid (LA), an essential omega-6 polyunsaturated fatty acid (PUFA) that the human body cannot synthesize (*11*, *12*). LA binding stabilizes S in a compact, locked conformation that is incompatible with ACE2 receptor binding (*11*). In immunofluorescence assays, synthetic mini-virus particles decorated with LA-bound S showed reduced docking to ACE2-expressing host cells as compared to mini-virus with LA-free S (*13*), corroborating that LA interferes with receptor binding and subsequent host cell entry mediated by S. S protein sequence alignments suggest conservation of the hydrophobic pocket in the RBDs of SARS-CoV, SARS-CoV-2, MERS-CoV, and the corresponding B domains in hCoV-OC43 and hCoV-HKU1 (*11*), indicating that the pocket may be a hallmark shared by all human β-CoVs. Intriguingly, all SARS-CoV-2 VOCs stringently maintain this pocket, notably including Omicron, which accumulated a wide range of mutations in S elsewhere, suggesting that the pocket provides a selective advantage for the virus.

Here, we sought to unveil whether LA binding and the functional consequences of LA binding are conserved in S glycoproteins of pathogenic β-CoVs SARS-CoV, MERS-CoV, and SARS-CoV-2 VOCs (Alpha, Beta, Gamma, Delta, and Omicron), as compared to HCoV-HKU1, a β-CoV causing only mild disease (common cold). We demonstrate that all comprise a hydrophobic pocket capable of binding LA, except common cold–causing HCoV-HKU1 S that cannot bind LA. At the same time, we demonstrate that a single–amino acid substitution of a residue lining the entrance of the hydrophobic pocket in HCoV-HKU1 S is sufficient to restore LA binding. We analyze SARS-CoV S by cryogenic electron microscopy (cryo-EM) showing that LA-bound SARS-CoV S adopts a hitherto elusive locked structure sharing characteristics with LA-bound locked SARS-CoV-2 S (*11*), incompatible with ACE2 receptor binding. In contrast, in the open conformation of SARS-CoV S, the pocket in the RBDs is devoid of LA. Molecular dynamics (MD) simulations corroborate spontaneous LA binding in the respective hydrophobic pockets in the RBDs of SARS-CoV, MERS-CoV, and SARS-CoV-2 VOCs, while no LA binding to HCoV-HKU1 S is observed. Using correlative light EM (CLEM) followed by electron tomography of SARS-CoV-2–infected cells, we provide evidence that LA, beyond counteracting infection at the S protein level (*11*, *13*), also interferes with viral replication inside infected cells. This likely occurs through inhibition of cytoplasmic phospholipase A2 (cPLA2), a key enzyme implicated in viral replication via formation of intracellular replication compartments (*14*) and in the cytokine storm causing systemic inflammation in COVID-19 (*15*–*17*).

## RESULTS

### Functional conservation of the S FFA-binding pocket in β-CoVs

**Fig. 1.**
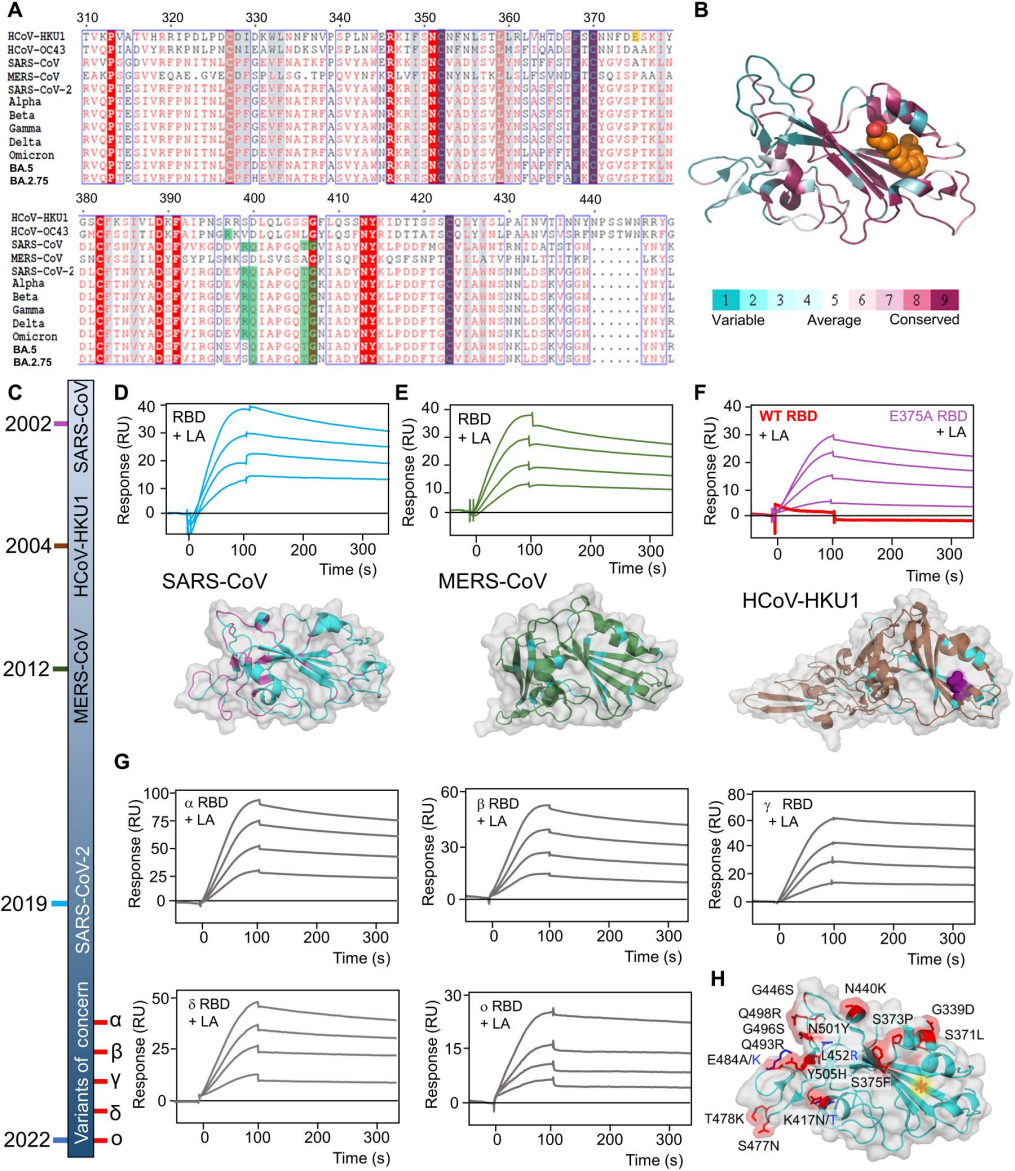
Conservation of LA-binding pocket in β-CoVs. (**A**) Alignments of the B domain of β-CoVs HCoV-HKU1 and HCoV-OC43, and the RBD of SARS-CoV, MERS-CoV, SARS-CoV-2, and VOCs (Alpha, Beta, Gamma, Delta, and Omicron), including the current BA.5 and BA.2.75 variants. Gray box: FFA pocket residues; green: residues in the hydrophilic lid; orange: residue E375 in HCoV-HKU1. (**B**) Conservation of residues within the domains is shown, calculated with ConSurf (*58*), mapped on the structure of SARS-CoV-2 RBD (PDB-ID 6ZB5). Orange spheres: LA. (**C**) Timeline (not proportional) illustrating the emergence of β-CoVs starting with SARS-CoV in 2002 up to SARS-CoV-2 VOC Omicron in 2022. Greek letters, VOC denominations. (**D** to **F**) SPR analysis of LA binding to immobilized SARS-CoV RBD (D), MERS-CoV RBD (E), HCoV-HKU1 wild type (WT), and E375A mutant B domain (F), and respective three-dimensional (3D) structures drawn in ribbon presentation. Residues shared with the SARS-CoV-2 RBD are colored in cyan. Purple spheres: E375 in HCoV-HKU1. (**G**) LA binding to RBDs from the five VOCs analyzed by SPR. LA concentrations ranging from 4 to 10 μM were used for all SPR experiments. For HCoV-HKU1 wild type, only 10 μM was tested. (**H**) Mutations found in the VOCs mapped on the 3D structure of SARS-CoV-2 Omicron RBD. Mutations are indicated. Orange asterisk: FFA-binding pocket; marine blue: mutations different from the Omicron variant sequence.

LA binding to S can be analyzed by surface plasmon resonance (SPR). We previously determined a binding constant of ~41 nM for LA to SARS-CoV-2 S RBD (*11*). To corroborate our hypothesis that a functional hydrophobic pocket is evolutionarily conserved, we tested whether other β-CoVs are also capable of LA binding (Fig. 1). On the basis of sequence alignments, the RBDs of SARS-CoV, MERS-CoV, SARS-CoV-2, and VOCs (Alpha, Beta, Gamma, Delta, and Omicron), including current BA.5 and BA.2.75 variants, all maintain the hydrophobic pocket, at least since the emergence of SARS-CoV in 2002 (Fig. 1, A to C). In SPR experiments, LA bound to immobilized SARS-CoV RBD (Fig. 1D). We observed a slow dissociation of LA from the RBD consistent with tight LA binding. LA also bound to immobilized MERS-CoV RBD (Fig. 1E). In contrast, the B domain of HCoV-HKU1 S did not bind LA despite high sequence similarity (Fig. 1, A and F). HCoV-HKU1 S comprises a bulky glutamate E375 located directly in front of the hydrophobic pocket (*18*), obstructing the pocket entrance (Fig. 1F). We mutated HCoV-HKU1 E375 to alanine and restored LA binding (Fig. 1F). This indicates that the pocket function, while structurally conserved, may have been lost in HCoV-HKU1, a β-CoV that causes mild disease. The RBDs of SARS-CoV-2 VOCs all bound LA, confirming that LA binding is conserved (Fig. 1G) and not affected by the mutations in S that cluster away from the pocket (Fig. 1H). Together, we confirmed full conservation of LA binding in highly pathogenic β-CoV S proteins but not in S of mild disease–causing HCoV-HKU1. HCoV-OC43, which likewise causes common cold, appears to also comprise a hydrophobic pocket (Fig. 1A), as seen in an earlier HCoV-OC43 S cryo-EM structure that displays unassigned density in the B domain (fig. S1) (*19*). It remains unclear what exactly this unassigned density corresponds to, which appears too small to accommodate the C18 hydrocarbon chain of LA (fig. S1).

### Cryo-EM structures of locked and open SARS-CoV S

**Fig. 2.**
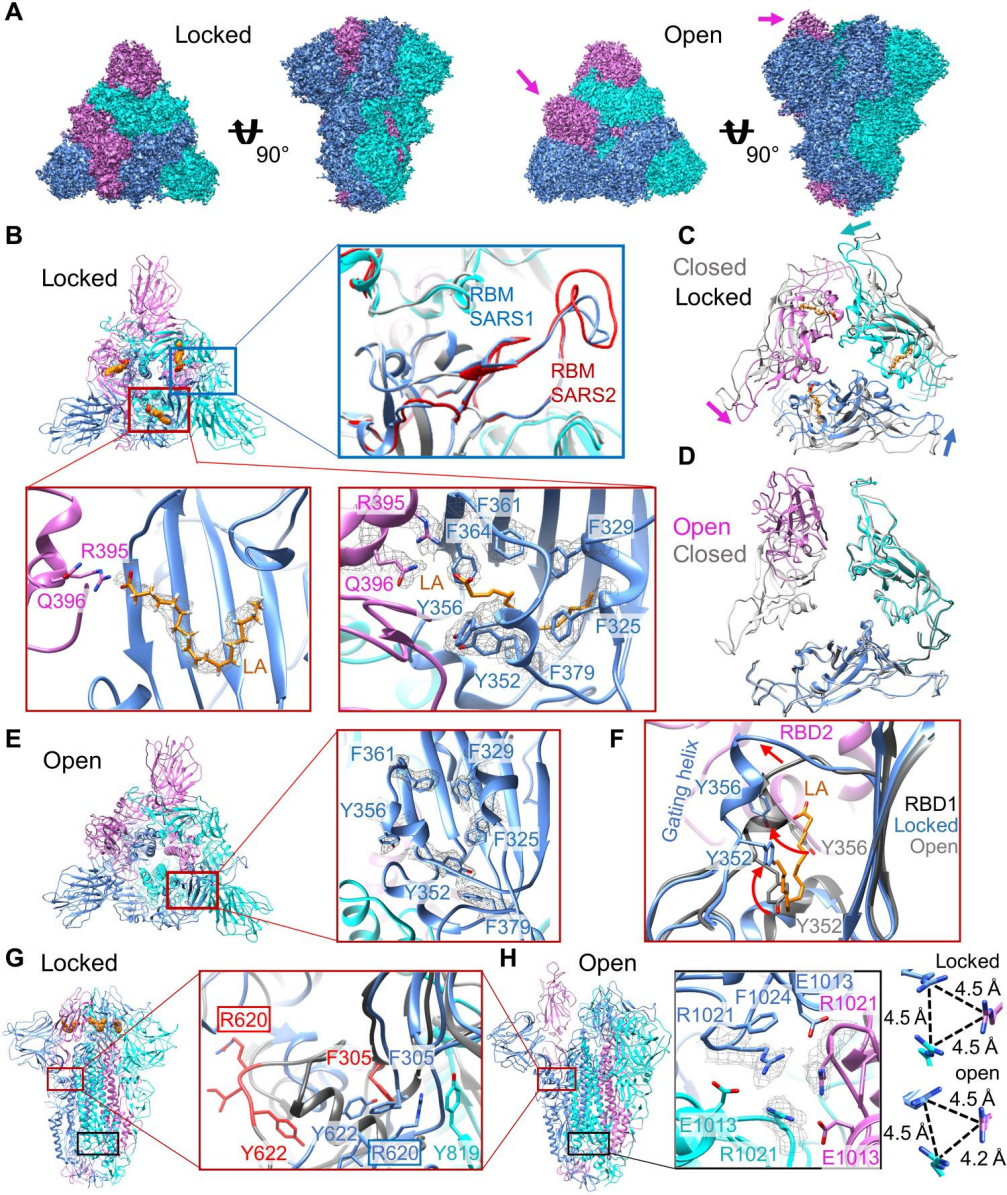
SARS-CoV S adopts LA-bound locked and LA-free open conformations. (**A**) Cryo-EM density of SARS-CoV S in locked (left) and open (right) conformations, front and top views. Monomers are in cyan, blue, and magenta. RBD-up conformation marked by arrows. (**B**) Top view of locked S, cartoon representation. Orange spheres: LA. Blue box: fully ordered RBM (blue) and SARS-CoV-2 S RBM (red) (PDB ID 6ZB5). Red boxes: LA-binding pocket formed by adjacent S RBDs. Left: LA’s headgroup interacts with R395 and Q396. Right: Density (mesh) for hydrophobic residues lining the pocket. (**C**) LA-bound S RBD trimers overlaid with SARS-CoV S, closed conformation without LA (gray) (PDB ID 6ACC). (**D**) Overlay of S RBD trimers, open and closed conformation (gray) (PDB ID 6ACC). (**E**) Open conformation, top view. Right: Zoom-in with density for hydrophobic residues lining the pocket (mesh). (**F**) Overlay of LA-bound and unbound S RBD from locked (blue) and open (gray) conformation. Red arrows: movement of gating helix, Y352 and Y356 upon LA binding. (**G**) Locked conformation, side view. Red box: region around R620. Close-up: open conformation (gray), residues R620-Y622 and F305 in red. Locked conformation: R620 (blue) stabilized by F305 (blue) and Y819 (cyan) from neighboring subunit. (**H**) Side view of open conformation. Black box: region around R1021, forming the center of an H-bond cluster, cation-π interactions with F1024, and salt bridge to E1013. Right: Short-range interactions of R1021 have threefold symmetry in locked conformation and are asymmetric in open conformation. Distances between the carbon of the arginine guanidinium group (CΖ atoms) indicated.

To elucidate LA binding by SARS-CoV that emerged 2002, we determined the S cryo-EM structure. The S ectodomain was produced as a secreted trimer using MultiBac (*20*) identically as described for SARS-CoV-2 S (fig. S2) (*11*). As before, we did not supplement LA during expression or subsequent sample purification and preparation steps. Cryo-EM data collection was performed with purified S protein (fig. S2 and table S1). Three-dimensional (3D) classification and refinement identified one conformation with all three RBDs in the down position and two different open conformations with one or two RBDs in the up position, respectively (fig. S3 and tables S1 and S2). Using 81,242 particles, the “one-RBD up” open conformation reached 3.3-Å resolution and was further analyzed (Fig. 2A and figs. S3 and S4). Analysis of 178,203 particles adopting the three RBD down conformation yielded a 2.48-Å resolution map after applying C3 symmetry (Fig. 2A and figs. S3 and S4). This three-RBD down form of SARS-CoV S exhibits a compact arrangement of the RBDs with fully ordered RBM, similar to our previously identified LA-bound locked S structure of SARS-CoV-2 (*11*).

Locked SARS-CoV S is stabilized by LA occupying a bipartite binding site composed of two adjacent RBDs in the trimer (Fig. 2, B and C). One RBD contributes a “greasy tube” lined by mostly phenylalanines accommodating the hydrocarbon tail of LA, and an adjacent RBD coordinates the polar headgroup of LA via residues R395 and Q396 (Fig. 2B). An overlay of the locked S structure with the previously determined LA-free “closed” structure of SARS-CoV S (Fig. 2C) (*21*) illustrates how LA binding induces compaction of the three RBDs, sharing characteristic features with locked SARS-CoV-2 S (*11*). Overlay of the one-RBD up open S conformation with the closed S conformation (*21*) shows that the down RBDs align very well (root mean square deviation = 1.086; Fig. 2D), while not aligning when overlaid with the RBDs in the locked S conformation. In agreement, we find no density for LA in the pocket of the down RBDs of this open S conformation, and the pocket appears collapsed (Fig. 2, E and F). The overlay of the RBDs from one chain in the open conformation with RBDs in the locked conformation illustrates rearrangements of Y352 and Y356 at the pocket entrance and a 5-Å movement of the gating helix to accommodate LA in the pocket (Fig. 2F). LA binding to S also induces a profound conformational change in the loop comprising residue R620, which then engages in a cation-π interaction with Y819 (Fig. 2G). Residue Y819 is part of the fusion peptide proximal region of the neighboring S subunit. This conformational rearrangement is completed by a π-π interaction of F305 and Y622 in the locked conformation (Fig. 2G). Another stabilization of the locked S trimer (and closed S trimer) is achieved by R1021 residues in each subunit, which form the center of a symmetric H-bond cluster, cation-π interactions with F1024, and salt bridges to E1013 (Fig. 2H). In the open conformation, this symmetry of the R1021 residue arrangement is broken, resulting in a destabilization of S (Fig. 2H). Together, LA-bound SARS-CoV S adopts a more stable, locked conformation incompatible with ACE2 binding, while the open conformation lacks LA and, as a consequence, is more flexible. We conclude that LA binding is fully conserved in SARS-causing coronaviruses since at least 2002.

### MD simulations of LA binding to β-CoV S FFA-binding pockets

**Fig. 3.**
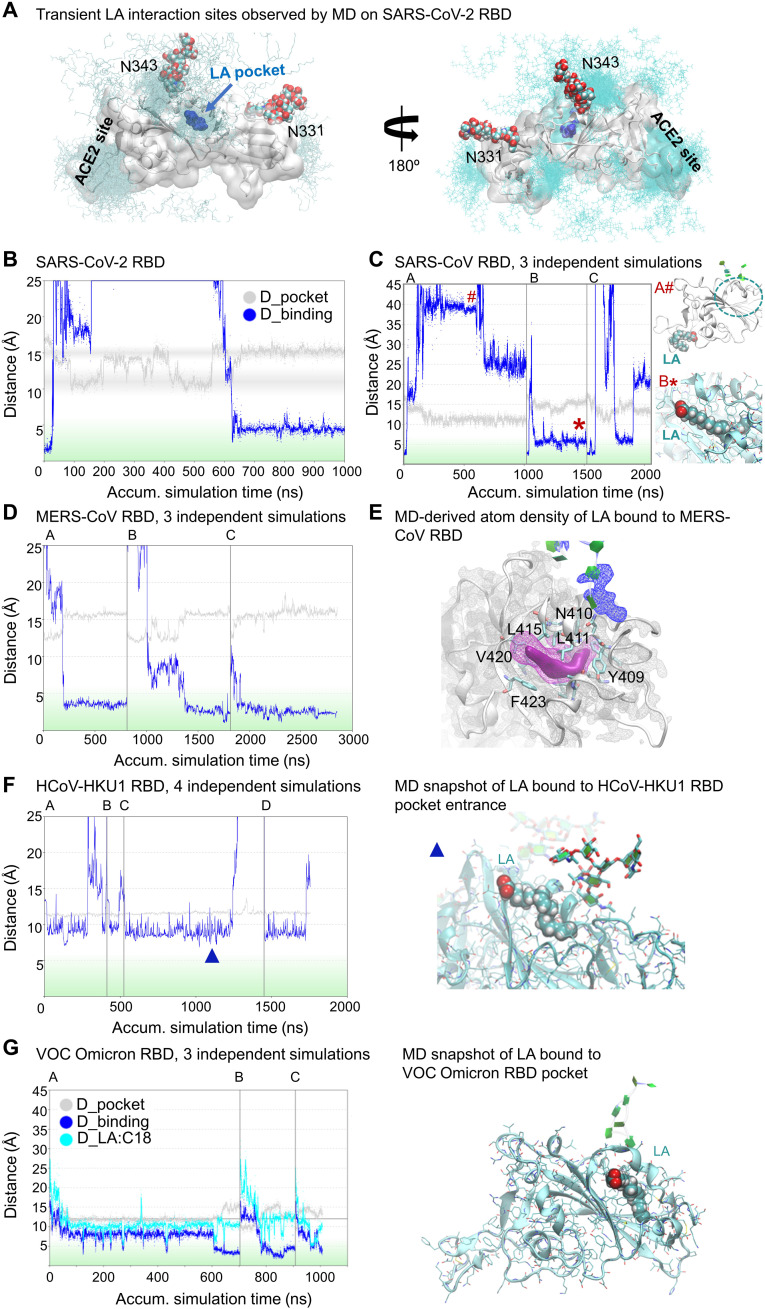
MD analysis of LA binding to β-CoV S proteins. (**A**) LA binding to the hydrophobic pocket of SARS-CoV-2 RBD [PDB ID 6zb5 (*11*)]. LA (cyan) transiently interacts with the RBD surface before entering the pocket (dark blue). Glycosylation at residues N343 and N331 is shown as spheres. LA positions correspond to first 600 ns of MD simulation shown in (**B**). Trajectories are colored identically in all panels. (B) Pocket dynamics (gray curve) and LA pocket binding (blue curve) to SARS-CoV-2 RBD (see also fig. S5, A and B). (**C**) LA binding to SARS-CoV RBD highlighting three different outcomes of simulations (A to C). Right: Position of LA during the simulation (on the surface and in the pocket indicated by # and *, respectively). (**D**) LA binding to MERS-CoV RBD, three simulations (A to C). (**E**) Atom-density iso-contour plot derived from accumulated 2.3-μs MD simulation of LA (magenta) bound to MERS-CoV RBD (gray, ribbon and density mesh; cyan, side chains; green, glycosylation groups). LA high atom density is also shown as solid iso-contour volume (magenta). (**F**) LA binding to HCoV-HKU1 B domain. Left: Four simulation trajectories (A to D). Right: LA binds at the pocket entrance during simulation (time point during simulation indicated by blue triangle). (**G**) LA binding to VOC Omicron RBD. Left: Three simulations (A to C) depicting pocket opening and binding by LA. Right: Snapshot from the end of the trajectory, with LA bound in the pocket.

Next, we scrutinized LA binding to the hydrophobic pockets of β-CoVs by extensive MD simulations (Fig. 3). As a proof of principle, unbiased and spontaneous LA binding to the SARS-CoV-2 RBD was simulated. Using the distance of the α-carbon atoms of residues N370 (gating helix) and F377 (pocket entrance) of the RBD, we monitored the dynamics of the opening and closing of the pocket during the simulations (D_pocket) (fig. S5A). As a measure of binding of LA in the pocket, we monitored the distance between the geometric center of all carbon and oxygen atoms of LA and the center of a set of atoms that are in contact with LA when bound in the hydrophobic pocket [as determined from Protein Data Bank (PDB) ID 6zb5] (D_binding) (fig. S5B). Starting with a bound state of LA [PDB ID 6zb5 (*11*)], the system was equilibrated for 30 ns, and then LA was pushed out of the pocket by applying a small force, which is indicated by a sharp increase of the distance of LA from the center of the pocket (Fig. 3B, blue curve). Without LA inside the pocket, the distance D_pocket fluctuates between open and closed states (gray curve changes from 15- to 10-Å distance; Fig. 3B) until LA (randomly) approaches the entrance after about 600 ns, binds back to the pocket, and stabilizes the open pocket (Fig. 3B and movie S1). Note that, during the MD simulation, LA rebinding to the pocket was not immediate. Instead, LA transiently interacted with residues on the surface of the SARS-CoV-2 RBD (simulation time from 40 to 600 ns in Fig. 3B and movie S1). We identified hotspots of LA interactions on the RBD surface, which include the RBM and glycosylated residue N343 and located close to the pocket entrance (Fig. 3A). LA binding on the RBD surface, including to the RBM and the neighborhood of residue N343 that is glycosylated, is transient and does not interfere detectably with LA binding to the pocket. Spontaneous (re)binding of LA to the hydrophobic pocket was observed once LA approached the pocket entrance (Fig. 3B and fig. S5C), and LA subsequently remained stably bound in the pocket.

Three different outcomes emerged in our MD simulations of LA (re)binding to the SARS-CoV RBD (Fig. 3C). (i) LA did not (re)enter the pocket during a 1-μs simulation but interacted closely with the RBM over a substantial time period (A in Fig. 3C); (ii) LA rebound to the pocket after removal (B in Fig. 3C and movie S2); and (iii) LA entered the pocket but could seemingly dissociate again (C in Fig. 3C). Additional simulations show that LA binding to the RBD is dynamic because the contacts formed between LA and the residues lining the pocket vary over time and between experiments, indicating diverse binding modes (fig. S6, A to D). However, when LA binding was analyzed for the complete SARS-CoV S in the MD simulations, LA was stably bound to the three pockets formed by adjacent RBDs within the S trimer with minimal dynamics (fig. S6, E and F), which is in line with tighter binding in the closed spike and more labile binding in the free RBD or open spike. After validating the simulation method with experimentally derived LA-bound RBD structures (Fig. 3, A to C), we applied the same MD simulation protocol to analyze LA binding to MERS-CoV RBD (from PDB ID 6q05). Spontaneous binding of LA to the pocket of MERS-CoV RBD was observed in 10 of 11 independent simulations (Fig. 3, D and E, and fig. S7A). Further analyses suggested a prevalent LA binding mode where LA does not entirely enter the hydrophobic pocket (fig. S7B) while demonstrating significant dynamics of the portion of LA within the pocket similar to SARS-CoV (fig. S7, C and D). Notably, LA binding and pocket opening occurred simultaneously in the MERS-CoV RBD, suggesting that LA can bind to the entrance of the closed pocket and pry open the gate (Fig. 3D and movie S3). As a control, we analyzed LA binding to the pocket in the HCoV-HKU1 B domain. LA was found to transiently interact with hydrophobic residues at the pocket entrance but did not enter the pocket in our simulations (Fig. 3F), reproducing our LA binding SPR experiments (Fig. 1C). In contrast, tight LA binding to the RBD pocket of SARS-CoV-2 VOC Omicron was observed, consistent with the SPR data (Fig. 3G and movie S4).

### LA treatment of SARS-CoV-2–infected cells suppresses viral replication

**Fig. 4.**
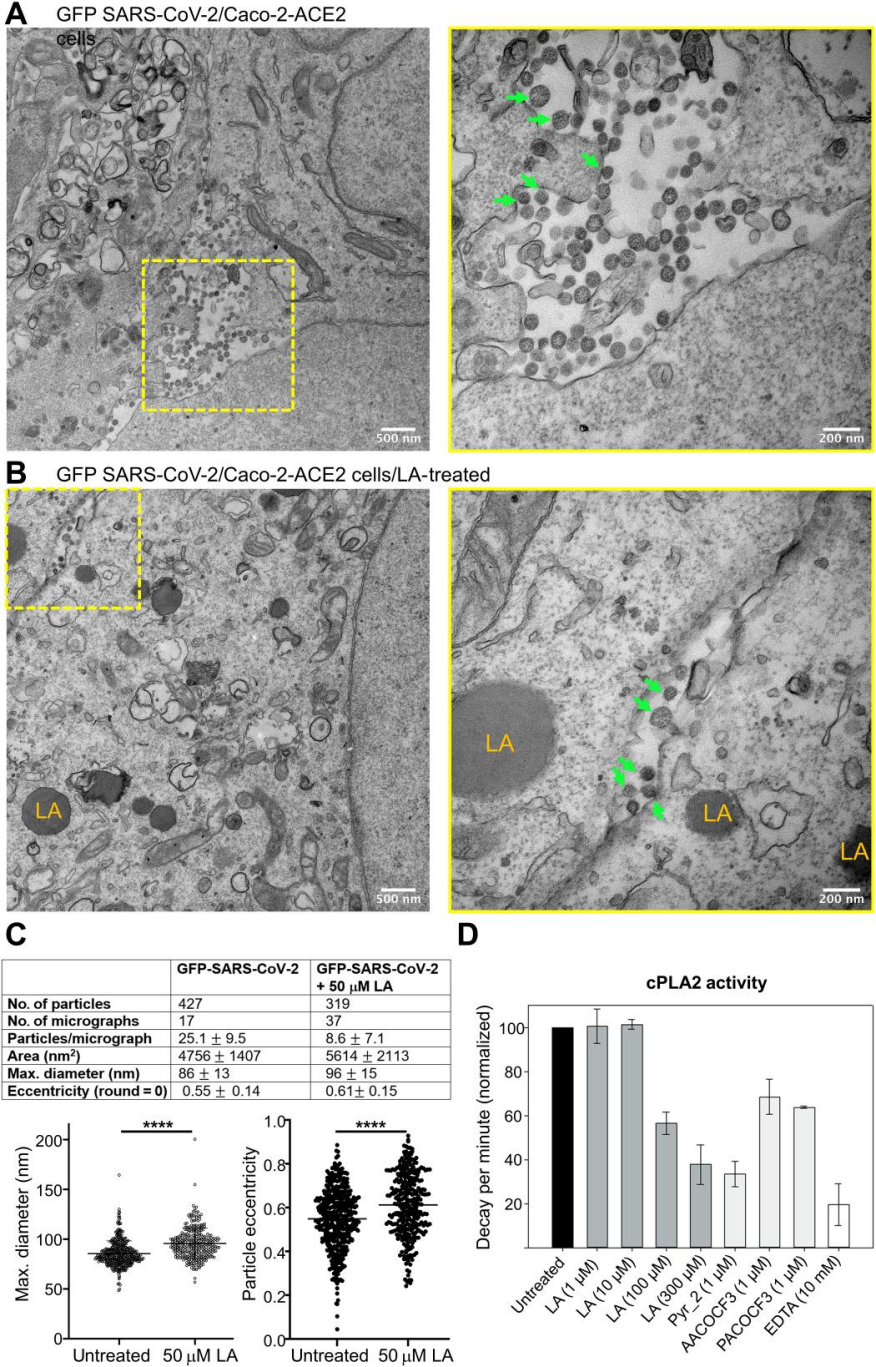
TEM analysis of GFP-expressing SARS-CoV-2–infected Caco-2-ACE2 cells. Transmission electron microscopy (TEM) images of cells in the absence (**A**) and presence (**B**) of treatment with 50 μM LA (added after infection). Left: Overview image of a GFP-SARS-CoV-2–infected cell. Yellow box highlights a region with virions. Scale bars, 500 nm. Right: Close-up view. Scale bars, 200 nm. Green arrows point to virions; LA highlights lipid droplets. (**C**) Particle quantification and size/shape analysis of virions from green cells. Values are indicated as means ± SD. Significance levels were determined by unpaired *t* test. ****P < 0.0001. (**D**) Dose-response inhibition of cPLA2 by LA, compared to cPLA2 inhibitors used as positive controls [Pyr_2 (pyrrolidine-2), AACOCF3, PACOCF3, and EDTA]. Experiments were repeated twice. Data are represented as disintegration per minute [% of maximal enzymatic activity measured in the absence of inhibitor (untreated)].

To evaluate the impact of LA treatment on SARS-CoV-2–infected cells, we infected Caco-2 cells overexpressing ACE2 (Caco-2-ACE2) with green fluorescent protein (GFP)–expressing SARS-CoV-2 and supplemented the cells with 50 μM LA (or solvent as a control) at 1 hour after infection. Viral replication and cell viability were monitored by bright-field and fluorescence microscopy. We used CLEM followed by electron tomography to analyze SARS-CoV-2–infected Caco-2-ACE2 cells 35 hours after LA supplementation (Fig. 4 and figs. S8 to S10). Despite analyzing only strong GFP-expressing cells, we detected significantly (*P* = 2.0 × 10^−6^) more virions in infected cells that were not LA-treated (~25 virions per micrograph) than in infected cells that had been treated with LA after infection (~9 virions per micrograph) (Fig. 4, A to C, and fig. S10). LA treatment also resulted in the emergence of lipid droplets in the cytoplasm of cells that appear dark in the EM micrographs (Fig. 4B and fig. S9). These droplets appear independent of whether the cells were SARS-CoV-2–infected or uninfected (Fig. 4B and figs. S9 and S10) and occur in many cells including adipocytes. Moreover, we notice significant membrane remodeling in SARS-CoV-2–infected Caco-2-ACE2 cells compared to noninfected cells, as reported previously for β-CoV infections (Fig. 4 and fig. S10) (*22*, *23*).

In addition to their reduced number (Fig. 4C), virions in SARS-CoV-2–infected, LA-treated cells appeared irregular in size and shape as compared to the virions in untreated cells that adopt a characteristic regular spherical form (Fig. 4, A and B; fig. S10; and movies S5 and S6). Closer analysis of virions derived from LA-treated cells confirmed a statistically significant increase in size and deformation (ellipticity) (Fig. 4C). The average diameter of virions from untreated cells (*n* = 427) was calculated as 86 nm (SD = 13 nm), in agreement with previous reports (*14*). In contrast, virions derived from LA-treated cells (*n* = 319) had a larger average diameter of 96 nm (SD = 15 nm). Consistently, the average area of virions increased from 4756 to 5614 nm^2^ (Fig. 4C). Moreover, the average ellipticity of virions increased from 0.55 (SD = 0.14) in untreated cells to 0.61 (SD = 0.15) in LA-treated cells, confirming deformation. Together, we observed that LA treatment after infection leads to a lower viral load in SARS-CoV-2–infected cells, with the virions being larger in size and deformed as compared to untreated, infected cells (Fig. 4, A to C), indicating that their integrity, and potentially their infectivity, may be compromised.

During early stages of coronavirus infection, PLA2s are activated as evidenced by increased cellular levels of lysophospholipids and FFAs such as LA, arachidonic acid, oleic acid, and palmitoleic acid in both SARS-CoV-2–infected cell cultures and SARS-CoV-2–infected patients (*16*, *24*–*26*). A central regulator of lipidome remodeling during β-CoV infection is cPLA2. Inhibition of this enzyme interferes with coronavirus-induced membrane rearrangements including formation of intracellular double-membrane vesicles and replicative organelles, which are essential for viral replication (*24*). cPLA2 cleaves glycerophospholipids at the *sn*-2 ester position, generating FFAs and lysophospholipids. Previously, it was shown that cPLA2 is tightly regulated by PUFAs, including LA, which are potent competitive inhibitors of the enzyme (*27*). We find that cPLA2 inhibition is half-maximal in the presence of ~100 μM LA in vitro (Fig. 4D). We note that LA binds S proteins of pathogenic β-CoVs with orders of magnitude higher affinity (Fig. 1) compared to cPLA2.

## DISCUSSION

On the basis of our findings, the conserved FFA-binding pocket emerges as a hallmark of pathogenic β-CoV S structure. The cryo-EM structure of SARS-CoV S demonstrates stringent conservation of the LA-binding pocket since at least 2002 when the SARS-CoV outbreak occurred (Fig. 2). We observe a high correlation between LA binding in the pocket and the locked conformation, which is a noninfectious form of S, incompatible with ACE2 receptor binding (Fig. 2B). MD simulations consistently show transient interactions of LA with hydrophobic patches on the surface of the RBDs before binding the pocket, suggesting a conserved mechanism of LA approaching the pocket entrance (Fig. 3). However, stable LA binding to the RBDs only occurs once LA has entered the pocket. It is less clear whether the pocket first has to open by moving the gating helix or whether LA binding at the entrance of the pocket can force open the pocket. We find examples for both scenarios in the different simulations, but an opening of the pocket while interacting with LA (induced fit) occurred more frequently (Fig. 3, B and D).

Previous studies showed that LA renders the virus less infectious by stabilizing a locked form of S inhibiting receptor binding, virion attachment, and entry into cells (*11*, *13*). Here, we show that LA treatment of cells that are already SARS-CoV-2–infected significantly reduces the production of virions. Moreover, the few virions produced are markedly deformed (Fig. 4, A to C, and fig. S10). These modes of action will likely synergize to significantly reduce, or even abrogate, viral infectivity and replication. Therefore, FFA binding by S can be conceived as an “Achilles’ heel” of pathogenic β-CoVs, making this feature a highly attractive target for LA- or LA mimetic–based antiviral interventions against SARS-CoV-2. Notably, fatty acids have been used prolifically as excipients in unrelated medications with established safety in multiple administration routes (nasal, pulmonary, oral, and intravenous) (*28*).

The evolutionary conservation of the FFA pocket implies significant selection advantage for the virus itself. We can conceive of several such advantages. For instance, the LA-bound S form is more stable than the open S forms (*29*). LA stabilizes the S protein in a locked, prefusion conformation (*12*), which is particularly useful when the protein is still inside the host cell or exits the host cell, preventing premature S opening, S1 shedding, and adoption of the postfusion conformation. Moreover, LA-bound, locked S buries key epitopes of the RBM and of core RBD parts, potentially hiding these from attack by neutralizing antibodies (*1*, *13*). We speculate that LA levels can be sensed by the virus, allowing LA to dissociate from the S protein and then switch from the preferred “stealth” locked form to open, infectious conformations that mediate ACE2 binding and cell entry. Lipid metabolome remodeling is a central element of viral infection (*25*, *30*, *31*). LA levels are markedly perturbed during COVID-19 disease progression, with serum levels significantly decreased in COVID-19 patients (*32*). Conversely, intracellular levels of LA are elevated (*25*). This correlates with β-CoV–induced membrane remodeling to generate new membrane compartments for viral replication (*22*, *23*). cPLA2 activation is a central mediator of lipidome remodeling in β-CoVs and of various additional +RNA virus families and is therefore a validated target for broad-spectrum antiviral drug development (*24*). We propose that, during β-CoV infection when cPLA2 is activated, the virus can circumvent feedback inhibition of cPLA2, e.g., by sequestering LA (Fig. 4D) (*27*). This would keep cPLA2 in a hyperactivated state. In this model, inhibition of cPLA2 by supplementing excess LA will down-regulate membrane remodeling, thus interfering with a mechanism required for viral replication. We show that LA treatment of SARS-CoV-2–infected cells interferes with viral replication (Fig. 4). Previous reports demonstrate that LA also interferes with MERS-CoV replication (*25*). In conclusion, our results convey that the conserved FFA-S interaction, while affording selective advantages to the virus, renders it vulnerable to antiviral intervention exploiting this highly conserved feature. This could be achieved by supplementing LA or a related molecule, ideally during early stages of infection when the virus resides in the upper respiratory tract where it can be conveniently targeted. This could be accomplished by delivering FFA formulations, e.g., via a nasal or inhaled spray, to suppress viral replication and spreading within a patient, concomitantly reducing transmission (*33*) and protecting others from infection, an unmet need and key element of COVID-19 treatment that remains elusive to date.

## MATERIALS AND METHODS

### Protein production

#### SARS-CoV S protein

The complementary DNA (cDNA) for expression of SARS-CoV S ectodomain (UniProt ID P59594, Isolate BJ01) was codon-optimized for insect cell expression and synthesized by GenScript Inc. (New Jersey, USA). In this construct, S ectodomain comprises amino acids 14 to 1193, preceded by the GP64 secretion signal sequence (amino acid sequence MVSAIVLYVLLAAAAHSAFA) at the N terminus. The construct is fused to a C-terminal thrombin cleavage site followed by a T4-foldon trimerization domain and a hexa-histidine affinity purification tag. The protein was expressed using the MultiBac baculovirus expression system (Geneva Biotech, Geneva, Switzerland) (*20*) in Hi5 cells using ESF921 medium (Expression Systems Inc.). Three days after transfection, the supernatant from transfected cells was harvested by centrifugation at 1000*g* for 10 min followed by a second centrifugation of the supernatant at 5000*g* for 30 min. The final supernatant was incubated with 5 ml of HisPur Ni-NTA Superflow Agarose (Thermo Fisher Scientific) per 2 liters of culture overnight at 4°C. A gravity flow column was used to collect the resin bound with SARS-CoV S protein. The resin was washed extensively with wash buffer [65 mM NaH_2_PO_4_, 300 mM NaCl, and 20 mM imidazole (pH 7.5)], and the protein was eluted using elution buffer [65 mM NaH_2_PO_4_, 300 mM NaCl, and 235 mM imidazole (pH 7.5)]. Elution fractions were analyzed by reducing SDS–polyacrylamide gel electrophoresis (PAGE), and fractions containing SARS-CoV S protein were pooled and concentrated using 50-kDa molecular weight cutoff Amicon centrifugal filter units (EMD Millipore) and buffer-exchanged in size exclusion chromatography (SEC) buffer [20 mM tris (pH 7.5) and 100 mM NaCl]. Concentrated SARS-CoV S was subjected to SEC using a Superdex 200 increase 10/300 column (GE Healthcare) in SEC buffer. Peak fractions from SEC were analyzed by reducing SDS-PAGE and negative-stain EM. Fraction 8 was used for cryo-EM (fig. S2).

#### Biotinylated SARS-CoV RBD

The SARS-CoV RBD-encoding DNA was codon-optimized for insect cell expression and synthesized by GenScript (New Jersey, USA). This construct comprised amino acid residues 306 to 527 and was fused at its N terminus to the SARS-CoV-2 S secretion signal sequence (amino acid sequence MFVFLVLLPLVSSQ) and was followed by a linker (amino acid sequence GGSGGSGSG), an avi-tag (amino acid sequence GLNDIFEAQKIEWHE), a second linker (amino acid sequence GSGSGS), and finally an octa-histidine tag for purification. This construct was inserted into pACEBac1 plasmid (Geneva Biotech, Geneva, Switzerland). The protein was produced and purified as described above for SARS-CoV S ectodomain except the last SEC step, which was performed in 1× phosphate-buffered saline (PBS; pH 7.5). Biotinylation was achieved by incubation with BirA in the presence of biotin according to established protocols (*34*). Remaining free biotin and BirA were removed by purifying biotinylated SARS-CoV RBD by SEC using an S200 10/300 increase column (GE Healthcare).

#### Biotinylated MERS-CoV RBD

The biotinylated MERS-CoV RBD expression construct was generated as described above for biotinylated SARS-CoV RBD. The construct comprises MERS-CoV (UniProt ID K0BRG7) residues 367 to 606. The protein was expressed and purified as described above.

#### Biotinylated HCoV-HKU1 B domain (wild type and E375A mutant)

Biotinylated HCoV-HKU1 B domain expression constructs were generated as described above for biotinylated SARS-CoV RBD. The wild-type construct contains HCoV-HKU1 (UniProt ID U3N885) residues 310 to 624. In E375A, glutamate 375 was mutated to alanine. The wild-type and E375A B domain proteins were expressed and purified as described above.

#### Biotinylated SARS-CoV-2 VOC RBDs

Biotinylated *SARS-CoV-2* RBDs from variant expression constructs were generated as described above for biotinylated SARS-CoV RBD. These constructs contain SARS-CoV-2 (UniProt ID P0DTC2) residues 319 to 541, with variant-specific mutations. The RBDs were expressed and purified as described above.

### Negative-stain sample preparation and EM

Four microliters of SARS-CoV S protein (0.05 mg/ml) was applied onto a freshly glow discharged (1 min at 10 mA) CF300-Cu-50 grid (Electron Microscopy Sciences), incubated for 1 min, and manually blotted. Four microliters of 3% uranyl acetate was applied onto the same grid and incubated for 1 min before the solution was blotted off. Images were acquired at a nominal magnification of ×49,000 on a FEI Tecnai 12 120-kV BioTwin Spirit microscope.

### Cryo-EM sample preparation and data collection

Four microliters of SARS-CoV S protein (0.5 mg/ml) was loaded onto a freshly glow discharged (2 min at 4 mA) Quantifoil R1.2/1.3 carbon grid (Agar Scientific), blotted using Vitrobot MarkIV (Thermo Fisher Scientific) at 100% humidity and 4°C for 2 s, and plunge-frozen. Data were acquired on a FEI Talos Arctica transmission electron microscope operated at 200 kV and equipped with a Gatan K2 Summit direct detector and Gatan Quantum GIF energy filter, operated in zero-loss mode with a slit width of 20 eV using the EPU software.

Data were collected in counted superresolution mode at a nominal magnification of ×130,000 with a physical pixel size of 1.05 Å/pixel and a virtual pixel size of 0.525 Å/pixel. The dose rate was adjusted to 5.77 counts per physical pixel per second. Each movie was fractionated in 60 frames of 200 ms. A total of 6600 micrographs were collected with a defocus range comprised between −0.8 and −2.0 μm.

### Cryo-EM data processing

The dose-fractionated movies were gain-normalized, aligned, and dose-weighted using MotionCor2 (*35*). Defocus values were estimated and corrected using the Gctf program (*36*). A total of 1,724,689 particles were automatically picked using Relion 3.0 (*37*). Reference-free 2D classification was performed to select well-defined particles. After three rounds of 2D classification, a total of 784,580 particles were selected for further 3D classification. The initial 3D model (*11*) was filtered to 60 Å during 3D classification in Relion using eight classes. Classes 4 and 5 (fig. S3), showing prominent features of closed conformation representing a total of 178,203 particles, were combined and used for 3D refinement. Class 6 comprised S proteins in a one-RBD up (open) conformation, and class 1 presented S in a two-RBD up (open) conformation, comprising 81,707 and 122,315 particles, respectively (fig. S3). The selected maps were subjected to 3D refinement without applying any symmetry. Subsequently, the maps were subjected to local defocus correction and Bayesian particle polishing in Relion 3.1. Global resolution and B factor (−68 and −100.8 Å^2^ for closed and open maps, respectively) of the maps were estimated by applying a soft mask around the protein density, using the gold standard Fourier shell correlation criterion 0.143, resulting in an overall resolution of 2.71 and 3.34 Å, respectively (fig. S4). C3 symmetry was applied to the closed conformation map using Relion 3.1, followed by CTF refinement and Bayesian polishing, yielding a final resolution of 2.48 Å (B factor of −73.71 Å^2^) (fig. S4). Local resolution maps were generated using Relion 3.1 (fig. S4).

### Cryo-EM model building and analysis

UCSF Chimera (*38*) was used to fit atomic models of the SARS-CoV S closed conformation [PDB ID 6ACC (*21*)] and open conformation [PDB ID 6ACD (*21*)] into our SARS-CoV C1 closed conformation and open conformation cryo-EM map, respectively. To improve the model building, we used the symmetrized C3 map for the closed conformation, as well as Namdinator (*39*) for open and closed conformations. Model building was done in Coot (*40*) with unsharpened and sharpened maps (*41*), and N-linked glycans were built into the density for all three models where visible (table S2). The RBD-up in the open conformation was fitted as a rigid body into the corresponding density because the resolution in this part of the EM structure is not sufficient to build an atomic model. Restraints for the LA were generated with eLBOW (*42*). The models for C1- and C3-symmetrized closed conformation and the open conformation were real space–refined with Phenix (*43*), and the quality was checked using MolProbity (*44*) and EMRinger (*45*). Figures were prepared using UCSF chimera and PyMOL (Schrodinger Inc.).

### SPR experiments

Interaction experiments using SPR between LA and different RBDs and B domains were carried out with a Biacore T200 system (GE Healthcare) according to the manufacturer’s protocols and recommendations and as described previously (*11*). Briefly, biotinylated proteins were immobilized on streptavidin-coated SA sensor chips at ~2500 resonance units (RUs). LA sodium salt was dissolved in PBS (pH 7.5) at a concentration of 10 mM and then serially diluted and injected at concentrations of 4, 6, 8, and 10 μM at a flow rate of 30 μl/min. The running buffer for all SPR measurements was PBS buffer (pH 7.5). All experiments were performed in triplicates.

### MD simulations

Starting structures for the simulations were set up using the graphical interface of YASARA (*46*). For SARS-CoV-2 RBD (residues 319 to 592), PDB ID 6zb5 (pocket open state, LA bound) (*11*) was used. Starting structures of SARS-CoV RBD (residues 307 to 577) were built on the basis of the structure reported here (pocket open state, LA bound). MERS-CoV RBD (residues 368 to 655) was modeled on the basis of PDB ID 6q05 or 4l3n (pocket closed state) (*47*, *48*). PDB ID 5gnb was used for HCoV-HKU1 B domain (residues 311 to 674) (*18*). In all MD simulations, glycan N343 was modeled as in https://glycosmos.org/glycans/show/G80858MF, in agreement with (*49*).

In general, the systems were solvated in 0.9% NaCl solution (150 mM), and simulations were performed at 310 K using periodic boundary conditions and using the AMBER14 force field. The box size was rescaled dynamically to maintain a water density of 0.996 g/ml. Simulations were performed using YASARA with GPU acceleration in “fast mode” (4-fs time step) (*50*) on “standard computing boxes,” e.g., equipped with one 12-core i9 CPU and NVIDIA GeForce GTX 1080 Ti.

Three types of molecular simulation protocols were performed, termed here “LA bound,” “LA unbinding,” and “LA de novo binding.” In the first and second protocols, the starting structure consists of an RBD/LA or B domain/LA complex that has the LA molecule bound in the experimentally determined binding pocket (“open pocket”). LA bound follows a standard NPT MD protocol. The second MD protocol (LA unbinding) consists of three periods: about 30-ns equilibration of the bound state, a short period where the LA was pushed out of the binding pocket by application of a force of 1 kcal/mol, and finally followed by a long MD sampling period in which the LA was free to diffuse through the bulk solvent or interact with the protein surface. The third MD protocol (LA de novo binding) aims to simulate LA binding “de novo” based on an experimentally determined RBD or B domain “apo” structure with a hidden LA pocket (“closed pocket”) and LA molecule(s) positioned in the bulk solvent. Although binding events of drug fragments have been simulated previously on special-purpose supercomputers designed specifically for MD simulations (*51*), the unbiased simulation of small-molecule binding events can now be still considered a “challenging endeavor” in computational chemistry.

During this project, in total, more than 150 MD trajectories were sampled starting with LA in either bound or unbound state: 36 for SARS-CoV-2 RBD wild type (15-μs accumulated time scale); 9 for SARS-CoV-2 K417N, E484K, and N501Y (5-μs accumulated time scale); 74 for SARS-CoV (20-μs accumulated time scale); 32 for MERS-CoV (11-μs accumulated time scale); and 9 for HCoV-HKU1 (4-μs accumulated time scale). Only scientific plots of the most relevant trajectories are shown to keep the complexity of the data presented reasonable. Further details can be found in the captions of figs. S5 to S7.

Conformational Analysis Tools (CAT; www.md-simulations.de/CAT/) was used for analysis of trajectory data, general data processing, and generation of scientific plots. VMD (*52*) was used to generate molecular graphics.

### Live SARS-CoV-2 experiments

#### Cells and virus propagation

A VeroE6 cell line modified to constitutively express the serine protease TMPRSS2 (Vero E6/TMPRSS2, obtained from the National Institute for Biological Standards and Control (NIBSC), UK) and the human gut epithelial cell line Caco-2 expressing ACE2 (Caco-2-ACE2, a gift of Y. Yamauchi, University of Bristol) were cultured at 37°C in 5% CO_2_ in Dulbecco’s modified Eagle’s medium (DMEM) and GlutaMAX (Gibco, Thermo Fisher Scientific) supplemented with 10% fetal bovine serum (FBS; Gibco, Thermo Fisher Scientific) and 0.1 mM nonessential amino acids (NEAA; Sigma Aldrich). A SARS-CoV-2 reporter virus expressing a gene encoding the fluorescent protein turboGFP in place of the ORF7 gene (termed rSARS-CoV-2/Wuhan/ORF7-tGFP) was generated using a SARS-CoV-2 (Wuhan isolate) reverse genetics system using the “transformation-associated recombination in yeast” approach (*53*). Eleven cDNA fragments with 70–base pair end-terminal overlaps that spanned the entire SARS-CoV-2 isolate Wuhan-Hu-1 genome (GenBank accession: NC_045512) and replaced ORF7 gene with the turboGFP gene were produced by GeneArt synthesis (Invitrogen, Thermo Fisher Scientific) as inserts in sequence-verified, stable plasmid clones. The 5′-terminal cDNA fragment was modified to contain a T7 RNA polymerase promoter and an extra “G” nucleotide immediately upstream of the SARS-CoV-2 5′ sequence, while the 3′-terminal cDNA fragment was modified such that the 3′ end of the SARS-CoV-2 genome was followed by a stretch of 33 “A”’s followed by the unique restriction enzyme site *Asc*I. The inserts were amplified by polymerase chain reaction using a Platinum SuperFi II mastermix (Thermo Fisher Scientific) and assembled into a full-length SARS-CoV-2 cDNA clone in the YAC vector pYESL1 using a GeneArt High-Order Genetic Assembly System (A13285, Invitrogen, Thermo Fisher Scientific) according to the manufacturer’s instructions. RNA transcripts produced from the YAC clone by transcription with T7 polymerase were used to recover infectious virus. Whole-genome sequencing confirmed the virus sequence. The virus was propagated in VeroE6/TMPRSS cells grown in infection medium [Eagle’s minimum essential medium plus GlutaMAX (MEM; Gibco) supplemented with 2% FBS and NEAA]. Cells were incubated at 37°C in 5% CO_2_ until cytopathic effects were observed, at which time the supernatant was harvested and filtered through a 0.2-mm filter, aliquoted, and stored at −80°C.

#### Viral detection by fluorescence

Caco-2-ACE2 cells were seeded onto 9-mm glass coverslips coated in a finder pattern of evaporated carbon (~10 nm) and poly-d-lysine in 24-well plates or in μClear 96-well Microplates (Greiner Bio-One) in DMEM supplemented with 10% FBS until cell coverage on the coverslips reached 25%. The cells were inoculated with rSARS-CoV-2/Wuhan/ORF7-tGFP at a multiplicity of infection of 5 in infection medium for 60 min at room temperature before the medium was removed and replaced with infection medium containing 50 μM LA and 0.25% dimethyl sulfoxide (DMSO) or 0.25% DMSO only. Control wells were treated the same but received no infectious inoculum. Cells were incubated at 37°C in 5% CO_2_ for 36 hours until turboGFP expression was detectable in cells in the 96-well plate by fluorescence imaging with the ImageXpress Pico Automated Cell Imaging System (Molecular Devices). Samples were inactivated and fixed by submersion in 4% paraformaldehyde for 60 min at room temperature. All work with infectious recombinant SARS-CoV-2 was done inside a class III microbiological safety cabinet in a containment level 3 facility at the University of Bristol.

#### Immunofluorescence analysis

Fixed coverslips were stained with 4′,6-diamidino-2-phenylindole (1 mg/ml) for 5 min and transferred to a 24-well imaging plate with an ultrathin (25-mm) film bottom (Eppendorf) containing 500 µl of PBS. Images were acquired on a Leica SP5II Acousto-Optical Beam Splitter confocal laser scanning microscope attached to an inverted DMI600 epifluorescence microscope using a 10× dry objective [numerical aperture (NA) of 0.3] and a 63× oil immersion objective (NA of 1.4). Low-magnification overviews of coverslips were acquired to identify location of region of interest on carbon finder pattern.

#### Transmission EM sample preparation

Following fluorescence imaging, coverslips were rinsed in 0.1 M sodium cacodylate buffer (pH 7.4) and postfixed in 2.5% glutaraldehyde in 0.1 M sodium cacodylate buffer at 4°C until further processing. Samples were subsequently stained and further cross-linked with osmium-ferrocyanide [1% OsO_4_, 1.5% K_4_Fe(CN)_6_.3H_2_O, and 0.1 M sodium cacodylate buffer] for 1 hour at 4°C, before en bloc staining in 3% uranyl acetate for 30 min. Following a dehydration series at room temperature in ethanol (70, 80, 90, 96, and 100%), coverslips were infiltrated with 50% epoxy resin (Agar Scientific) in propylene oxide for 1 hour, followed by 100% epoxy resin two times for 30 min. Coverslips with cells facing up were covered in fresh epoxy resin and polymerized at 60°C for 48 hours. After ~16 hours, an epoxy resin stub was placed on top of the coverslip and the samples were returned to the oven. Coverslips were removed from blocks using liquid nitrogen and boiling H_2_O to reveal the carbon finder pattern. The blocks were trimmed to the region of interest and sectioned using a UC6 Leica ultramicrotome with a diamond knife (Diatome). Ultrathin sections (70 nm) were collected onto pioloform-coated slot grids and poststained with uranyl acetate and lead citrate for 10 and 4 min, respectively, before imaging at 120 kV using a Tecnai 12 BioTwin Spirit TEM. Virus particles were measured in images acquired at ×18,500 magnification in Fiji, as described (*54*).

#### Electron tomography

Sections (300 nm) collected on Pioloform-coated slot grids (Agar Scientific) were incubated in a solution of 15-nm gold fiducial markers (Aurion) for 5 min on each side. Tilt series (−65° to +65° at 1.5° increments) were acquired at ×19,000 magnification (0.5261 nm/pixel) using a FEI Tecnai 20 transmission electron microscope operated at 200 kV and equipped with a 4k-by-4k FEI Eagle camera. Electron tomograms were reconstructed using fiducial markers for alignment in IMOD (*55*).

### cPLA2 activity assays

Recombinant human cytosolic PLA2 (cPLA2 group IVA, PLA2G4A) was expressed and purified from insect cells as described (*56*). The enzymatic activity of cPLA2 was measured using *Escherichia coli* membranes radiolabeled with [^3^H]-oleic acid as described (*57*). To measure the inhibitory effect of LA, recombinant human cPLA2 (10 nM final concentration) was preincubated in the absence or presence of various concentrations of LA (1 to 300 μM) for 15 min in 100 μl of PLA2 activity buffer [100 mM tris-HCl (pH 8.0), 10 mM CaCl_2_, and 0.1% bovine serum albumin (BSA)], after which 30,000 dpm of radiolabeled *E. coli* membranes was added (diluted in 100 μl of buffer), with further incubation for 1 hour at 37°C. Enzymatic reactions were stopped by addition of 1 volume (200 μl) of PLA2 stop buffer (100 mM EDTA and 0.2% fatty acid–free BSA). Tubes were centrifuged at 14,000 rpm for 5 min, and supernatant containing released [^3^H]-oleic acid bound to BSA was collected and counted in a Tri-Carb liquid scintillation counter (PerkinElmer). The inhibitory effect of LA on cPLA2 was compared with that of AACOCF3 (Cayman Chemicals, #62120), PACOCF3 (Cayman Chemicals, #62650), and pyrrolidine-2 (Calbiochem, #525143) under the same assay conditions.

## Supplementary Materials

### This PDF file includes:

Figs. S1 to S10 Tables S1 and S2 References

## Notes

View/request a protocol for this paper from *Bio-protocol*.
