# Supplementary material for: The free fatty acid–binding pocket is a conserved hallmark in pathogenic β-coronavirus spike proteins from SARS-CoV to Omicron
Source: Sci Adv. 2022 Nov 23;8(47):eadc9179. doi: 10.1126/sciadv.adc9179 (PMC9683698; doi:10.1126/sciadv.adc9179)
Supplement: Supplementary file 1 — Figs. S1 to S10 Tables S1 and S2 References [file sciadv.adc9179_sm.pdf]

Science Advances
AAAS

Supplementary Materials for

The free fatty acid–binding pocket is a conserved hallmark in pathogenic β-coronavirus spike proteins from SARS-CoV to Omicron

Christine Toelzer *et al.*

Corresponding author: Martin Frank, martin.frank@biognos.se; Imre Berger, imre.berger@bristol.ac.uk; Christiane Schaffitzel, cb14941@bristol.ac.uk

Sci. Adv. **8**, eadc9179 (2022)
DOI: 10.1126/sciadv.adc9179

The PDF file includes:

Figs. S1 to S10
Tables S1 and S2
Legends for movies S1 to S6
References

Other Supplementary Material for this manuscript includes the following:

Movies S1 to S6

Fig. S1

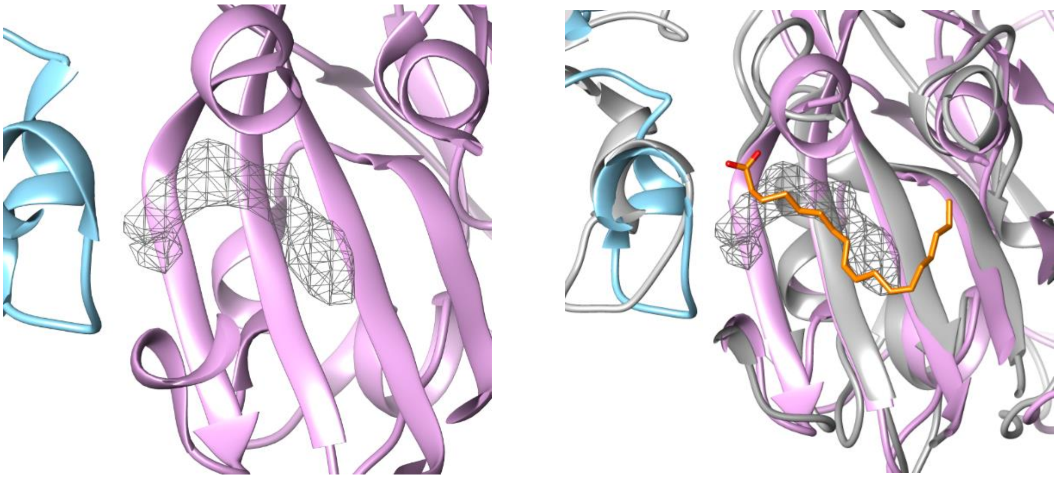

Unassigned density in the cryo-EM structure of HCoV-OC43. On the left, a zoomed view of the interface of two B domains of HCoV-OC43 S is shown (PDB ID 6OHW). HCoV-OC43 B domains are colored in blue and magenta. Unassigned density is shown as a mesh. A superimposition with the structure of locked SARS-CoV-2 S (PDB ID 6ZB5) in the same view is shown on the right. SARS-CoV-2 RBDs are colored in grey. LA is colored in orange, with oxygens of the polar head group colored in red.

Fig. S2

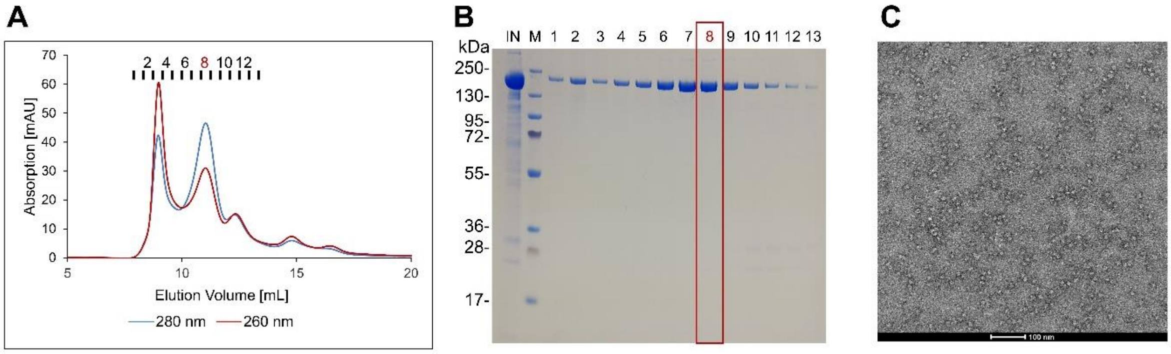

Purification and quality control of SARS-CoV S protein. (**A**) Size-exclusion chromatogram of affinity-purified SARS-CoV S protein using a Superdex 200 column. Absorption was detected at 280 nm (blue line) and 260 nm (red line). Peak fractions are indicated. The first peak at 8.6 mL elution volume corresponds to the void volume of the column comprising nucleic acids and S protein as confirmed by the SDS-PAGE analysis in panel B (fractions 1 and 2). The S trimer elutes at 11 mL. (**B**) SDS PAGE analysis of the SEC fractions from panel A. Lane 1: input fraction, lane 2: molecular weight marker, lane 3-15: fractions 1 to 13 from SEC. (**C**) Negative-stain EM micrograph of SEC peak fraction 8 (scale bar: 100 nm). In panels A and B peak fraction 8 is highlighted; this fraction was used for negative-stain EM and cryo-EM sample preparations.

Fig. S3

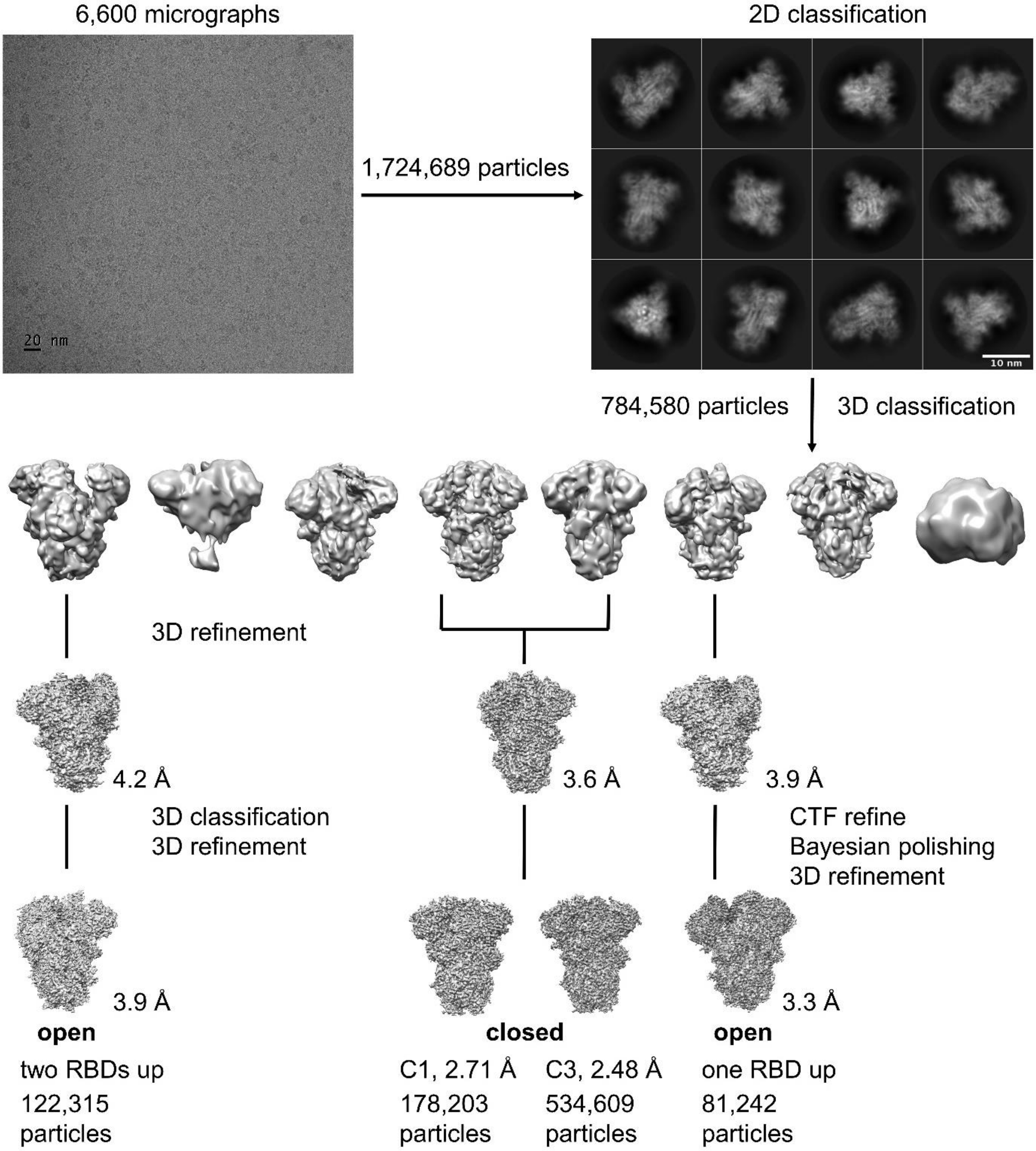

Cryo-EM image processing workflow. A motion-corrected cryo-EM micrograph (scale bar 20 nm), reference-free 2D class averages (scale bar 10 nm), 3D classification and refinement resulting in cryo-EM maps corresponding to the open conformations and the closed conformation (not symmetrized (C1) and C3-symmetrized) are shown.

Fig. S4

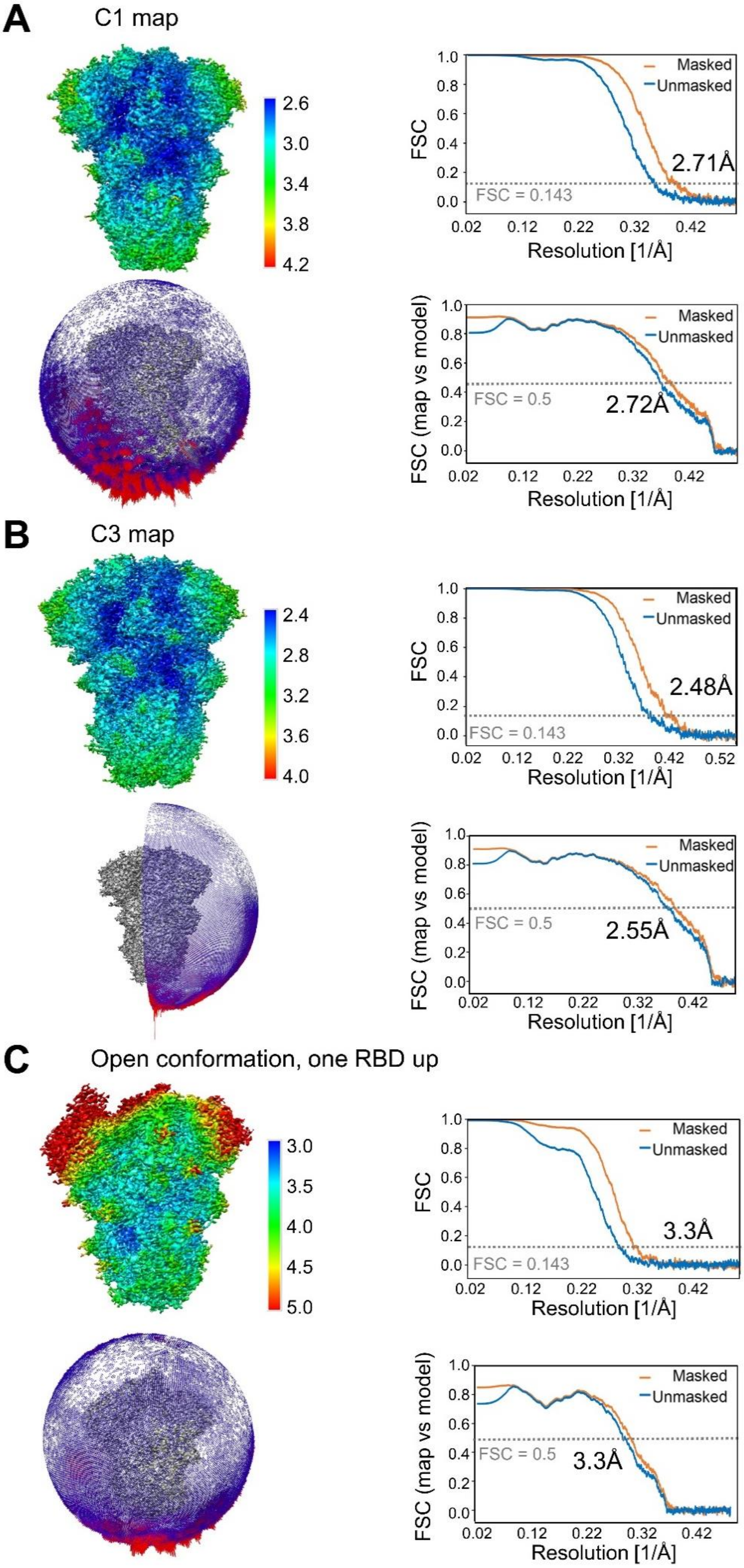
A
C1 map
2.6
3.0
3.4
3.8
4.2
FSC
Masked
Unmasked
2.71Å
FSC = 0.143
Resolution [1/Å]
FSC (map vs model)
FSC = 0.5
2.72Å
B
C3 map
2.4
2.8
3.2
3.6
4.0
2.48Å
2.55Å
C
Open conformation, one RBD up
3.0
3.5
4.0
4.5
5.0
3.3Å
3.3Å

Cryo-EM structure validation. Above left: Cryo-EM reconstruction colored according to the local resolution from a side view. Above right: Fourier Shell Correlation (FSC) curve after gold standard refinement. Below left: Orientation distribution of views that contributed to this map. Longer red rods represent orientations that comprise more particles. Below right: Cross-validation FSC curves for the refined model versus the final masked and unmasked maps. Corresponding panels are shown for (**A**) the closed unsymmetrized C1 map (**B**) the closed C3-symmetrized map and (**C**) the open, one RBD up conformation map.

Fig. S5

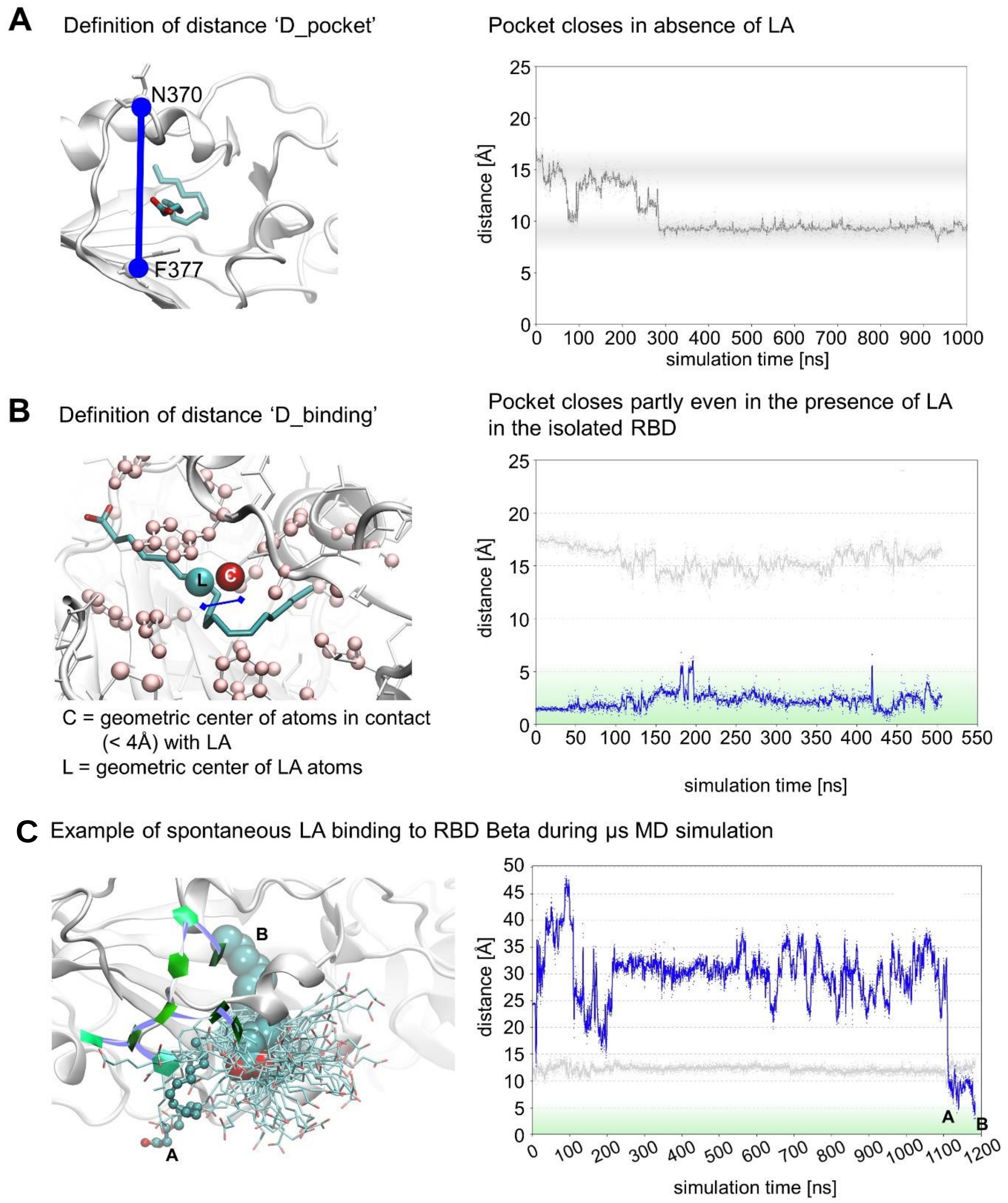

MD analysis of pocket dynamics and LA-binding to SARS-CoV-2 S RBD. (**A**) D_pocket (grey curve, right) is the distance between the Cα atoms of residues Asn370 and Phe377) shown as blue spheres in the RBD structure on the left (PDB ID 6ZGE (*59*)). D_pocket thus monitors the dynamic opening and closing of the pocket, as visualized on the right side. Open and closed states are indicated by gray bars at ~15 Å and ~9 Å, respectively. After eliminating LA, the pocket closes, indicated by a decrease of D_pocket as shown on the right. (**B**) LA

dynamics measured by parameter D_binding. C is the geometric center of the pocket. Left panel: D_binding is the distance between the center of the LA molecule (L) and C. L and C are shown as cyan and red spheres in the RBD structure (PDB ID 6ZGE). Right panel: Plotted are both D_binding (blue curve) and D_pocket (grey curve), monitoring the binding and dynamics of LA in the pocket (or in the case of HCoV-HKU1 to a hydrophobic site on the surface of the B domain) and pocket opening, respectively. The green bar indicates distances compatible with LA-binding in the pocket. In the isolated LA-bound RDB, the pocket entrance, as measured by D_pocket (grey curve), appears to be slightly smaller than in the locked S trimer. (**C**) An additional successful binding trajectory is shown for the B.1.351/Beta VOC (see also Fig 3B). Here, the pocket was closed in the starting structure. Left panel: The binding event (time between A and B) is represented as a sequence of overlayed MD snapshots to show that LA entry into the pocket is highly dynamic. LA is shown in teal stick representations, with time point A as small spheres, time point B as large spheres, and all other snapshots as stick only and with oxygens of the polar head group colored in red. Right panel: D_pocket (grey curve) and D_binding (blue curve) distances are shown.

Fig. S6

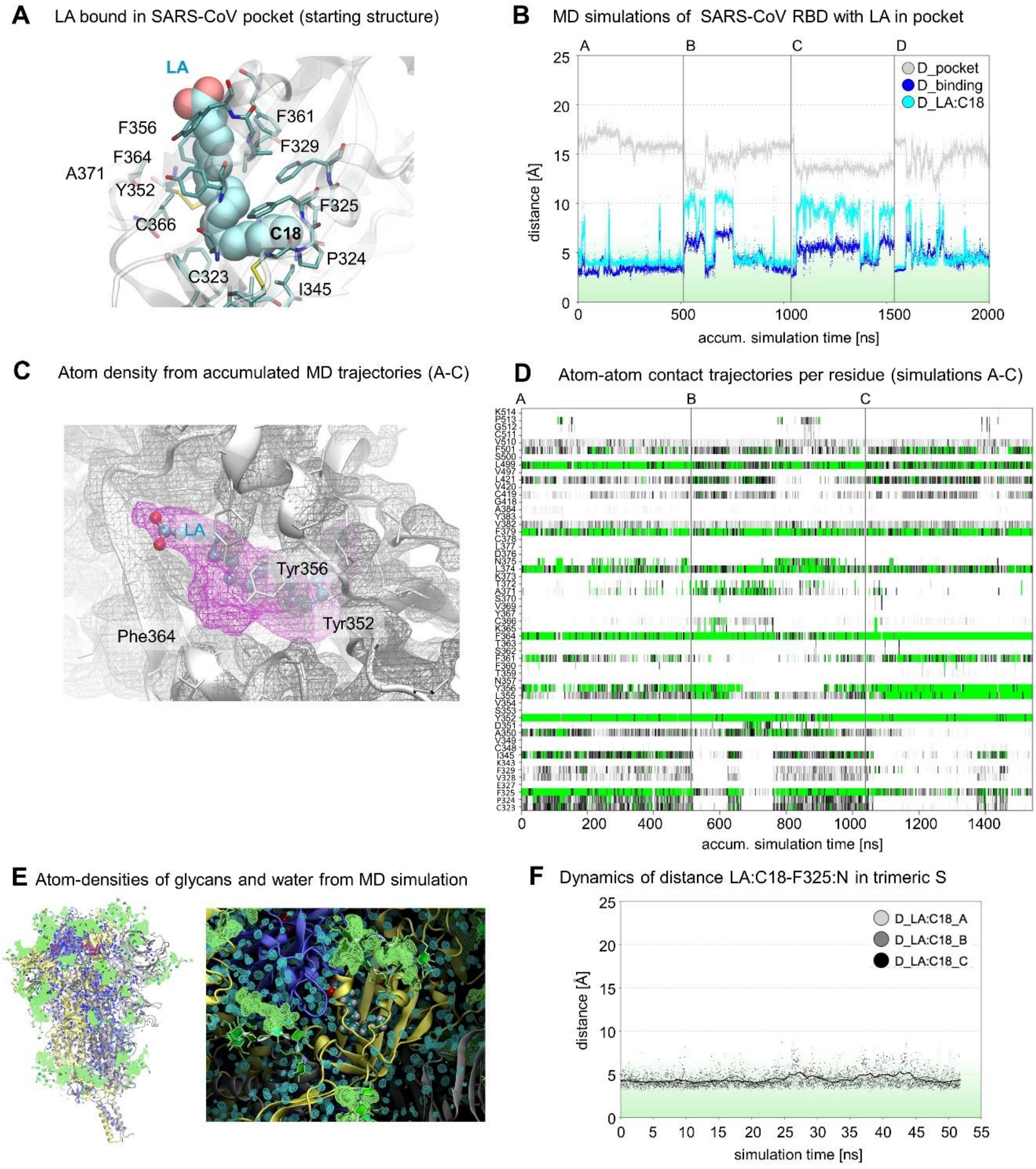

MD analysis of dynamic LA-binding to SARS-CoV S RBD. (**A**) Starting SARS-CoV S RBD structure with LA bound in the pocket (this study). Hydrophobic residues forming the pocket and the C18 atom of LA are labeled. (**B**) 2 µs MD analysis with LA 'stably' bound in the pocket of isolated RBD. However, the C18 atom of LA (cyan curve) can move away from the sub-pocket formed by F325, P324, I345 and C323 (see structure in panel A). Trajectories for D-pocket (grey curve), D_binding (blue curve) and D_LA:C18 (cyan curve, being a measure of the distance between the C18 atom of LA and the geometric center of the pocket) are plotted. (**C**) The dynamic LA-binding in the pocket is shown as density (pink mesh) derived from the

accumulated MD trajectories A-C shown in panel B. (**D**) Number of atom-atom contacts (<4 Å) of residues forming the LA-binding pocket (Y-axis) with LA, derived from the MD trajectories A-C in panel B. In green (corresponding to 10 atom-atom contacts or more) the dynamics of strong interactions are highlighted, *e.g.,* with F325, Y352, Y356, F364, L374, F379 and L499. (**E**) Analysis of LA-binding to SARS-CoV S. Left panel: A side view of a single MD snapshot of the SARS-CoV S (protein chains are depicted in grey, yellow and blue cartoon representation). Right panel: A zoomed view of the LA-binding pocket. LA represented as cyan spheres with hydrogens and carbons in white and red respectively and glycans as sticks in light green. In the closed/locked SARS-CoV S trimer, LA appears rigidly positioned in a single binding mode. (**F**) Analysis of the dynamics of LA in the pocket (D_LA:C18). The distance LA:C18-F325:N is shown in 50 ns MD simulations of the trimeric S for the three LA-pockets (in RBD chains A, B and C), confirming that LA is firmly bound in all three pockets. D_pocket and D_binding are defined in fig. S5A,B.

Fig. S7

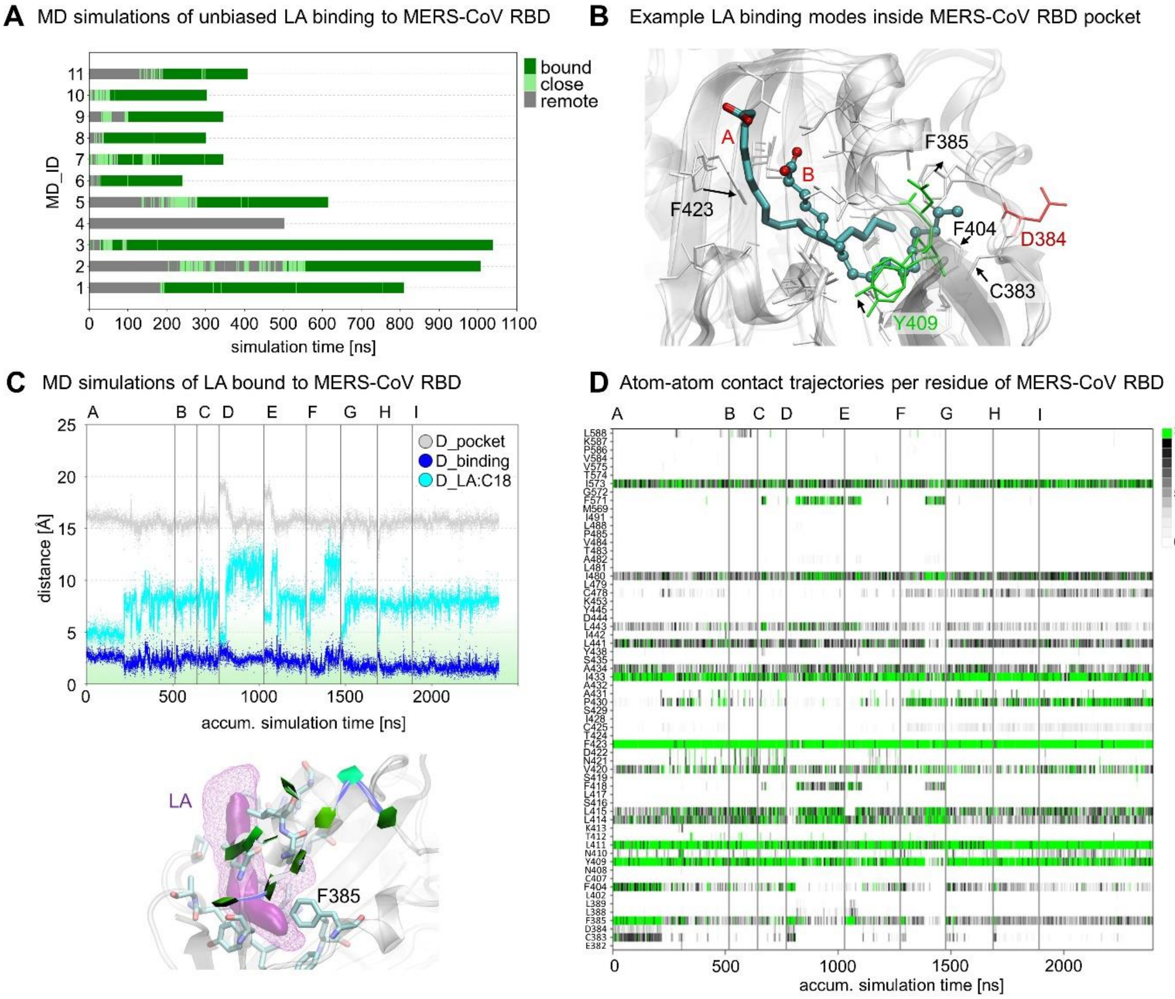

LA-binding to the pocket in the RBD of MERS-CoV S. (**A**) 11 MD trajectories are shown for LA-binding to the pocket in MERS-CoV RBD. Bound (LA in the pocket), close (LA at pocket entrance) and remote states are shown in green, light green and grey, respectively. In 10 simulations, spontaneous LA-binding (green) was observed in less than 500 ns. (**B**) Following the MD simulations, LA is binding in the pocket mainly in mode A (with a distance D_LA:C18 of about 8 Å, LA is represented as cyan sticks). However, in analogy to SARS-CoV S MD simulations (fig. S6), a binding mode B (with C18 of LA bound to a pocket formed by Phe385, Asp384, Cys383 and Phe404, LA is represented as cyan spheres) may be also plausible. (**C**) Above: Multiple MD trajectories of LA bound to the MERS-CoV RBD. The different distances D_pocket (grey curve), D_binding (blue curve) and D_LA:C18 (cyan curve) are shown as accumulated trajectory plot. Below: the LA atom density calculated from the accumulated MDs is shown as iso-contour plot in pink (solid: high atom density; mesh: lower atom density) within the MERS-CoV S RBD. LA-binding inside the pocket is stable. However, the short distance (<5Å) between LA:C18 and F385:N is not maintained after the restraints are

released. (**D**) Number of atom-atom contacts (<4 Å) of LA with residues forming the LA-binding pocket (Y-axis) in the MERS-CoV S RBD, derived from MD simulations (panel C). Strong interactions (10 or more contacts) are depicted in green.

Fig. S8

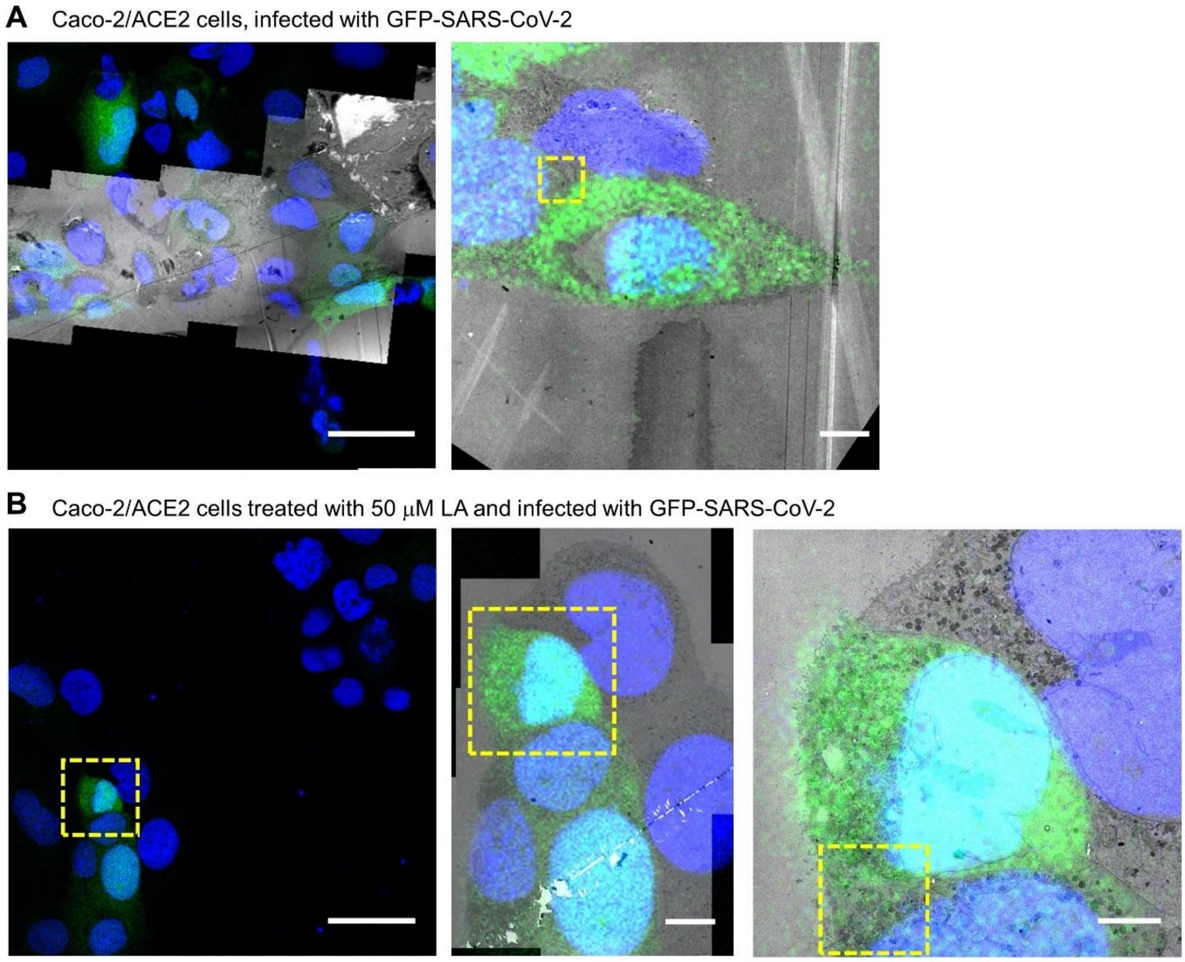

Correlative Light-Electron Microscopy (CLEM) of GFP-expressing SARS-CoV-2 infected Caco-2-ACE2 cells. (**A**) Overlay of fluorescence microscopy image and TEM mosaic. Left: Overview image. Scale bar (50 μm) colored in white. The yellow box highlights the region of interest used for further analysis. Right: Close-up view of the cells chosen for further analysis. The yellow box highlights the region shown in Fig. 4A which was used for electron tomography. Blue indicates DAPI staining of nuclei and green the GFP-SARS-CoV-2 virus. Scale bar (5 μm) colored in white. (**B**) Left: overview image from fluorescence microscopy for Caco-2-ACE2 cells infected with GFP-SARS-CoV-2 virus (green) and treated with 50 μM LA 1-hour after infection. Scale bar (50 μm) is colored in white. A green infected cell was chosen for further analysis. Middle and right image: Close-up views of the SARS-CoV-2 infected cell. The yellow box (left image) highlights the region shown in Fig. 4B which was used for electron tomography. Scale bar (5 μm) colored in white.

Fig. S9

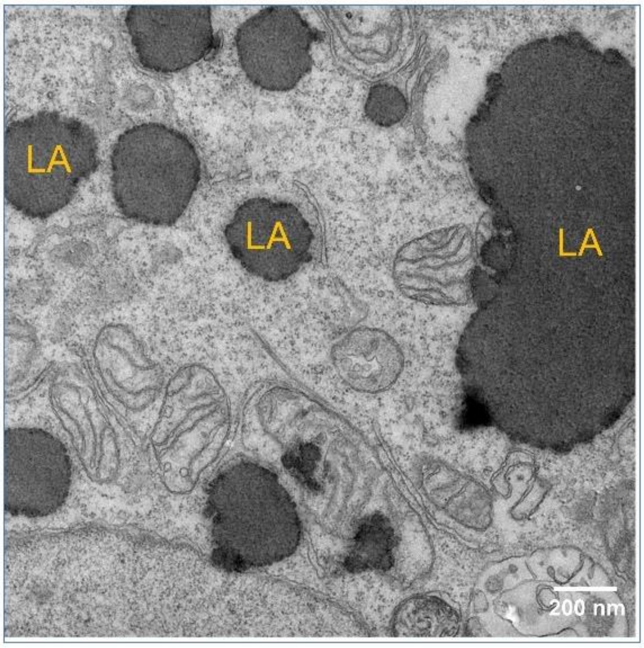

Uninfected Caco-2-ACE2 cell treated with LA. LA is readily taken up by the cells and appears as high contrast regions (marked with LA). The scale bar (200 nm) is colored in white.

Fig. S10

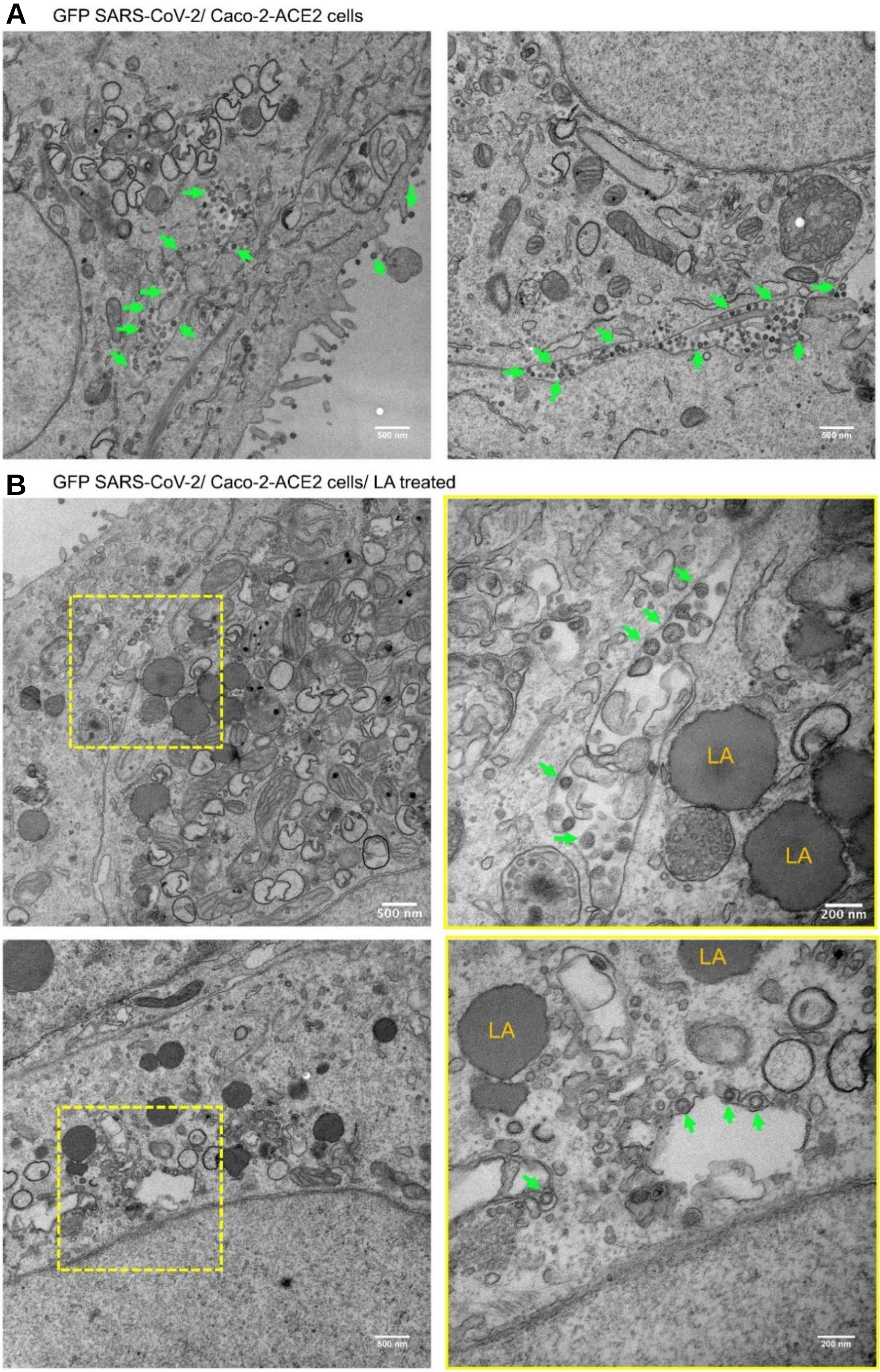

A
GFP SARS-CoV-2/ Caco-2-ACE2 cells
500 nm
500 nm
B
GFP SARS-CoV-2/ Caco-2-ACE2 cells/ LA treated
500 nm
LA
LA
200 nm
500 nm
LA
LA
200 nm

EM analysis of GFP-expressing SARS-CoV-2 infected Caco-2-ACE2 cells. Only green (GFP-expressing) cells were analyzed. Scale bar: 500 nm. (**A**) Image of two different cells in the absence of LA treatment. Green arrows point to virions. (**B**) Left: Overview images of GFP-SARS-CoV-2 infected cells treated with 50 μM LA after infection. Scale bar: 500 nm. Yellow boxes highlight regions with virions. Right: Close-up views. Scale bar: 200 nm. Green arrows point to virions. LA highlights lipid droplets.

Table S1

Cryo-EM data collection and refinement statistics for SARS-CoV.

	**Closed conformation, C3 symmetrized**	**Closed conformation, C1**	**Open conformation, C1**
Voltage (kV)	200	200	200
Magnification (nominal)	130,000	130,000	130,000
Pixel size (Å/pix)	1.05 (0.525)	1.05 (0.525)	1.05 (0.525)
Flux (e^- /pix/sec)	5.77	5.77	5.77
Frames per exposure	60	60	60
Exposure (e^- /Å^2)	1.04	1.04	1.04
Defocus range (μm)	-0.8 to -2.0	-0.8 to -2.0	-0.8 to -2.0
Micrographs collected	6603	6603	6603
Particles, final	534,609	178,203	81,242
Map sharpening B-factor (Å^2)	-73.71	-68.0	-100.8
Masked resolution at 0.143 FSC (Å)	2.48 Å	2.71 Å	3.3 Å

Refinement

	**Closed conformation, C3 symmetrized**	**Closed conformation, C1**	**Open conformation, C1**
Composition			
Amino acids	3060	3060	2785
Glycans	36	33	25
Ligands	3	3	-
RMSD bonds (Å)	0.004	0.004	0.006
RMSD angles (º)	0.617	0.586	0.671
Mean B-factors (Å^2)			
Amino acids	30.44	22.76	25.56
Ligands	28.52	29.97	40.26
Ramachandran			
Favored (%)	96.32	96.29	89.15
Allowed (%)	3.48	3.68	10.74
Outliers (%)	0.2	0.03	0.11
Rotamer outliers (%)	2.19	2.35	4.49
Clash score	2.28	2.85	5.65
C-beta outliers (%)	0.00	0	0.00
CaBLAM outliers (%)	3.66	3.53	6.37
CC (mask)	0.79	0.81	0.76
MolProbity score	1.51	1.60	2.37
EMRinger score	3.85	3.82	2.5
Model resolution (Å) 0.5 FSC threshold	2.6	2.9	3.3

Table S2
N-linked glycosylation sites in the SARS-CoV S protein

**SARS-CoV S**	
**WT* predicted**	**Recombinant, expressed in Hi5**
**UNIPROT (P59594)**	**this study ****
$N_{29}YT$	
$N_{65}VT$	$N_{65}VT$
$N_{73}HT$	
$N_{109}KS$	$N_{109}KS$
$N_{118}NS$	
$N_{119}ST$	$N_{119}ST$
$N_{158}CT$	$N_{158}CT$
$N_{227}IT$	$N_{227}IT$
$N_{269}GT$	$N_{269}GT$
$N_{318}IT$	$N_{318}IT$
$N_{330}AT$	$N_{330}AT$
$N_{357}ST$	$N_{357}ST$
$N_{589}AS$	
$N_{602}CT$	$N_{602}CT$
$N_{691}NT$	$N_{691}NT$
$N_{699}FS$	$N_{699}FS$
$N_{783}FS$	$N_{783}FS$
$N_{1056}FT$	$N_{1056}FT$
$N_{1080}GT$	$N_{1080}GT$
$N_{1116}NT$	
$N_{1140}HT$	
$N_{1155}AS$	
$N_{1176}ES$	

***** *NC_004718.3 (Isolate BJ01) (SARS-CoV)*
***Sites lacking glycosylation in cryo-EM maps are omitted (boxes colored in grey)*

Movies Captions

Movie S1: Simulation of LA-binding to the SARS-CoV-2 RBD (PDB ID 6ZB5 (*11*)) viewed from the pocket entrance. LA is shown as spheres colored teal for carbon and red for the oxygen in the carboxyl headgroup.

Movie S2: Simulation of LA-binding to the pocket in SARS-CoV RBD (this study), viewed from the pocket entrance and then from inside the pocket. LA is shown as spheres colored teal for carbon and red for the oxygen in the carboxyl headgroup.

Movie S3: Simulation of LA-binding to the pocket in MERS-CoV RBD (PDB ID 6Q05 (*47*), with closed pocket), viewed from the pocket entrance. LA is shown as spheres colored teal for carbon and red for the oxygen in the carboxyl headgroup.

Movie S4: Simulation of LA-binding to the pocket in the SARS-CoV-2 Omicron RBD (from PDB ID 7OAQ (*60*), with closed pocket), viewed from the pocket entrance. LA is shown as spheres colored teal for carbon and red for the oxygen in the carboxyl headgroup.

Movie S5: Digital sections (z = 1.05 nm, total depth 220 nm) through an electron tomogram of a SARS-CoV-2-infected Caco-2-ACE2 cell at 36 hrs after infection, as shown in Fig. 4A. To facilitate image alignment during image reconstruction, a suspension of 15-nm gold particles was layered on each side of the sections as fiducial markers

Movie S6: Digital sections (z = 1.05 nm, total depth 233 nm) through an electron tomogram of a SARS-CoV-2 infected Caco-2-ACE2 cell at 36 hours after infection, treated with 50 μM LA 1-hour after infection, as shown in Fig. 4B. To facilitate image alignment during image reconstruction, a suspension of 15-nm gold particles was layered on each side of the sections as fiducial markers.

The tomography movies S5 and S6 were taken at 19000x corresponding to a pixel size of 0.5261 nm/px. Data was binned during the processing resulting in a final pixel value of 1.05nm/pixel.

REFERENCES AND NOTES

1. K. Tao, P. L. Tzou, J. Nouhin, R. K. Gupta, T. de Oliveira, S. L. Kosakovsky Pond, D. Fera, R. W. Shafer, The biological and clinical significance of emerging SARS-CoV-2 variants. *Nat. Rev. Genet.* **22**, 757–773 (2021).

2. A. Rambaut, E. C. Holmes, Á. O'Toole, V. Hill, J. T. McCrone, C. Ruis, L. du Plessis, O. G. Pybus, Addendum: A dynamic nomenclature proposal for SARS-CoV-2 lineages to assist genomic epidemiology. *Nat. Microbiol.* **6**, 415 (2021).

3. L. Du, Y. He, Y. Zhou, S. Liu, B.-J. Zheng, S. Jiang, The spike protein of SARS-CoV—A target for vaccine and therapeutic development. *Nat. Rev. Microbiol.* **7**, 226–236 (2009).

4. M. Hoffmann, H. Kleine-Weber, S. Schroeder, N. Krüger, T. Herrler, S. Erichsen, T. S. Schiergens, G. Herrler, N.-H. Wu, A. Nitsche, M. A. Müller, C. Drosten, S. Pöhlmann, SARS-CoV-2 cell entry depends on ACE2 and TMPRSS2 and is blocked by a clinically proven protease inhibitor. *Cell* **181**, 271–280.e8 (2020).

5. M. Letko, A. Marzi, V. Munster, Functional assessment of cell entry and receptor usage for SARS-CoV-2 and other lineage B betacoronaviruses. *Nat. Microbiol.* **5**, 562–569 (2020).

6. A. C. Walls, Y.-J. Park, M. A. Tortorici, A. Wall, A. T. McGuire, D. Veesler, Structure, function, and antigenicity of the SARS-CoV-2 spike glycoprotein. *Cell* **181**, 281–292.e6 (2020).

7. L. Du, Y. Yang, Y. Zhou, L. Lu, F. Li, S. Jiang, MERS-CoV spike protein: A key target for antivirals. *Expert Opin. Ther. Targets* **21**, 131–143 (2017).

8. R. J. G. Hulswit, Y. Lang, M. J. G. Bakkers, W. Li, Z. Li, A. Schouten, B. Ophorst, F. J. M. van Kuppeveld, G. J. Boons, B. J. Bosch, E. G. Huizinga, R. J. de Groot, Human coronaviruses OC43 and HKU1 bind to 9-O-acetylated sialic acids via a conserved receptor-binding site in spike protein domain A. *Proc. Natl. Acad. Sci. U.S.A.* **116**, 2681–2690 (2019).

9. D. Wrapp, N. Wang, K. S. Corbett, J. A. Goldsmith, C. L. Hsieh, O. Abiona, B. S. Graham, J. S. McLellan, Cryo-EM structure of the 2019-nCoV spike in the prefusion conformation. *Science* **367**, 1260–1263 (2020).

10. Y. Yuan, D. Cao, Y. Zhang, J. Ma, J. Qi, Q. Wang, G. Lu, Y. Wu, J. Yan, Y. Shi, X. Zhang, G. F. Gao, Cryo-EM structures of MERS-CoV and SARS-CoV spike glycoproteins reveal the dynamic receptor binding domains. *Nat. Commun.* **8**, 15092 (2017).

11. C. Toelzer, K. Gupta, S. K. N. Yadav, U. Borucu, A. D. Davidson, M. Kavanagh Williamson, D. K. Shoemark, F. Garzoni, O. Staufer, R. Milligan, J. Capin, A. J. Mulholland, J. Spatz, D. Fitzgerald, I. Berger, C. Schaffitzel, Free fatty acid binding pocket in the locked structure of SARS-CoV-2 spike protein. *Science* **370**, 725–730 (2020).

12. K. Gupta, C. Toelzer, M. K. Williamson, D. K. Shoemark, A. S. F. Oliveira, D. A. Matthews, A. Almuqrin, O. Staufer, S. K. N. Yadav, U. Borucu, F. Garzoni, D. Fitzgerald, J. Spatz, A. J. Mulholland, A. D. Davidson, C. Schaffitzel, I. Berger, Structural insights in cell-type specific evolution of intra-host diversity by SARS-CoV-2. *Nat. Commun.* **13**, 222 (2022).

13. O. Staufer, K. Gupta, J. E. Hernandez Bücher, F. Kohler, C. Sigl, G. Singh, K. Vasileiou, A. Yagüe Relimpio, M. Macher, S. Fabritz, H. Dietz, E. A. Cavalcanti Adam, C. Schaffitzel, A. Ruggieri, I. Platzman, I. Berger, J. P. Spatz, Synthetic virions reveal fatty acid-coupled adaptive immunogenicity of SARS-CoV-2 spike glycoprotein. *Nat. Commun.* **13**, 868 (2022).

14. S. Klein, M. Cortese, S.L. Winter, M. Wachsmuth-Melm, C.J. Neufeldt, B. Cerikan, M. L. Stanifer, S. Boulant, R. Bartenschlager, P. Chlanda, SARS-CoV-2 structure and replication characterized by in situ cryo-electron tomography. *Nat. Commun.* **11**, 5885 (2020).

15. A.-S. Archambault, Y. Zaid, V. Rakotoarivelo, C. Turcotte, É. Doré, I. Dubuc, C. Martin, O. Flamand, Y. Amar, A. Cheikh, H. Fares, A. E. Hassani, Y. Tijani, A. Côté, M. Laviolette, É. Boilard, L. Flamand, N. Flamand, High levels of eicosanoids and docosanoids in the lungs of intubated COVID-19 patients. *FASEB J.* **35**, e21666 (2021).

16. J. M. Snider, J. K. You, X. Wang, A. J. Snider, B. Hallmark, M. M. Zec, M. C. Seeds, S. Sergeant, L. Johnstone, Q. Wang, R. Sprissler, T. F. Carr, K. Lutrick, S. Parthasarathy, C. Bime, H. H. Zhang, C. Luberto, R. R. Kew, Y. A. Hannun, S. Guerra, C. E. McCall, G. Yao, M. del Poeta, F. H. Chilton, Group IIA secreted phospholipase A2 is associated with the pathobiology leading to COVID-19 mortality. *J. Clin. Invest.* **131**, e149236 (2021).

17. E. Barberis, S. Timo, E. Amede, V. V. Vanella, C. Puricelli, G. Cappellano, D. Raineri, M. G. Cittone, E. Rizzi, A. R. Pedrinelli, V. Vassia, F. G. Casciaro, S. Priora, I. Nerici, A. Galbiati, E. Hayden, M. Falasca, R. Vaschetto, P. P. Sainaghi, U. Dianzani, R. Rolla, A. Chiocchetti, G. Baldanzi, E. Marengo, M. Manfredi, Large-scale plasma analysis revealed new mechanisms and molecules associated with the host response to SARS-CoV-2. *Int. J. Mol. Sci.* **21**, 8623 (2020).

18. X. Ou, H. Guan, B. Qin, Z. Mu, J. A. Wojdyla, M. Wang, S. R. Dominguez, Z. Qian, S. Cui, Crystal structure of the receptor binding domain of the spike glycoprotein of human betacoronavirus HKU1. *Nat. Commun.* **8**, 15216 (2017).

19. M. A. Tortorici, A. C. Walls, Y. Lang, C. Wang, Z. Li, D. Koerhuis, G. J. Boons, B. J. Bosch, F. A. Rey, R. J. de Groot, D. Veesler, Structural basis for human coronavirus attachment to sialic acid receptors. *Nat. Struct. Mol. Biol.* **26**, 481–489 (2019).

20. D. J. Fitzgerald, P. Berger, C. Schaffitzel, K. Yamada, T. J. Richmond, I. Berger, Protein complex expression by using multigene baculoviral vectors. *Nat. Methods* **3**, 1021–1032 (2006).

21. W. Song, M. Gui, X. Wang, Y. Xiang, Cryo-EM structure of the SARS coronavirus spike glycoprotein in complex with its host cell receptor ACE2. *PLOS Pathog.* **14**, e1007236 (2018).

22. K. Knoops, M. Kikkert, S. H. E. Worm, J. C. Zevenhoven-Dobbe, Y. van der Meer, A. J. Koster, A. M. Mommaas, E. J. Snijder, SARS-coronavirus replication is supported by a reticulovesicular network of modified endoplasmic reticulum. *PLOS Biol.* **6**, e226 (2008).

23. L. Mendonça, A. Howe, J. B. Gilchrist, Y. Sheng, D. Sun, M. L. Knight, L. C. Zanetti-Domingues, B. Bateman, A.-S. Krebs, L. Chen, J. Radecke, V. D. Li, T. Ni, I. Kounatidis, M. A. Koronfel, M. Szynkiewicz, M. Harkiolaki, M. L. Martin-Fernandez, W. James, P. Zhang,

Correlative multi-scale cryo-imaging unveils SARS-CoV-2 assembly and egress. *Nat. Commun.* **12**, 4629 (2021).

24. C. Müller, M. Hardt, D. Schwudke, B. W. Neuman, S. Pleschka, J. Ziebuhr, Inhibition of cytosolic phospholipase A2α impairs an early step of coronavirus replication in cell culture. *J. Virol.* **92**, e01463-17 (2018).

25. B. Yan, H. Chu, D. Yang, K. H. Sze, P. M. Lai, S. Yuan, H. Shuai, Y. Wang, R. Y. T. Kao, J. F.W. Chan, K. Y. Yuen, Characterization of the lipidomic profile of human coronavirus-infected cells: Implications for lipid metabolism remodeling upon coronavirus replication. *Viruses* **11**, 73 (2019).

26. J. Pungerčar, F. Bihl, G. Lambeau, I. Križaj, What do secreted phospholipases A2 have to offer in combat against different viruses up to SARS-CoV-2? *Biochimie* **189**, 40–50 (2021).

27. L. R. Ballou, W. Y. Cheung, Inhibition of human platelet phospholipase A2 activity by unsaturated fatty acids. *Proc. Natl. Acad. Sci. U.S.A.* **82**, 371–375 (1985).

28. M. J. Hackett, J. L. Zaro, W. C. Shen, P. C. Guley, M. J. Cho, Fatty acids as therapeutic auxiliaries for oral and parenteral formulations. *Adv. Drug Deliv. Rev.* **65**, 1331–1339 (2013).

29. I. Berger, C. Schaffitzel, The SARS-CoV-2 spike protein: Balancing stability and infectivity. *Cell Res.* **30**, 1059–1060 (2020).

30. C. M. Goodwin, S. Xu, J. Munger, Stealing the keys to the kitchen: Viral manipulation of the host cell metabolic network. *Trends Microbiol.* **23**, 789–798 (2015).

31. I. Casari, M. Manfredi, P. Metharom, M. Falasca, Dissecting lipid metabolism alterations in SARS-CoV-2. *Prog. Lipid Res.* **82**, 101092 (2021).

32. B. Shen, X. Yi, Y. Sun, X. Bi, J. Du, C. Zhang, S. Quan, F. Zhang, R. Sun, L. Qian, W. Ge, W. Liu, S. Liang, H. Chen, Y. Zhang, J. Li, J. Xu, Z. He, B. Chen, J. Wang, H. Yan, Y. Zheng, D. Wang, J. Zhu, Z. Kong, Z. Kang, X. Liang, X. Ding, G. Ruan, N. Xiang, X. Cai, H. Gao, L. Li,

S. Li, Q. Xiao, T. Lu, Y. Zhu, H. Liu, H. Chen, T. Guo, Proteomic and metabolomic characterization of COVID-19 patient sera. *Cell* **182**, 59–72.e15 (2020).

33. S. Doaei, S. Gholami, S. Rastgoo, M. Gholamalizadeh, F. Bourbour, S. E. Bagheri, F. Samipoor, M. E. Akbari, M. Shadnoush, F. Ghorat, S. A. Mosavi Jarrahi, N. Ashouri Mirsadeghi, A. Hajipour, P. Joola, A. Moslem, M. O. Goodarzi, The effect of omega-3 fatty acid supplementation on clinical and biochemical parameters of critically ill patients with COVID-19: A randomized clinical trial. *J. Transl. Med.* **19**, 128 (2021).

34. M. Fairhead, M. Howarth, Site-specific biotinylation of purified proteins using BirA. *Methods Mol. Biol.* **1266**, 171–184 (2015).

35. S. Q. Zheng, E. Palovcak, J.P. Armache, K. A. Verba, Y. Cheng, D. A. Agard, MotionCor2: Anisotropic correction of beam-induced motion for improved cryo-electron microscopy. *Nat. Methods* **14**, 331–332 (2017).

36. K. Zhang, Gctf: Real-time CTF determination and correction. *J. Struct. Biol.* **193**, 1–12 (2016).

37. S. H. Scheres, RELION: Implementation of a Bayesian approach to cryo-EM structure determination. *J. Struct. Biol.* **180**, 519–530 (2012).

38. T. D. Goddard, C. C. Huang, T. E. Ferrin, Visualizing density maps with UCSF Chimera. *J. Struct. Biol.* **157**, 281–287 (2007).

39. R. T. Kidmose, J. Juhl, P. Nissen, T. Boesen, J. L. Karlsen, B. P. Pedersen, Namdinator - automatic molecular dynamics flexible fitting of structural models into cryo-EM and crystallography experimental maps. *IUCrJ* **6**, 526–531 (2019).

40. P. Emsley, B. Lohkamp, W. G. Scott, K. Cowtan, Features and development of Coot. *Acta Crystallogr. D Biol. Crystallogr.* **66**, 486–501 (2010).

41. T. C. Terwilliger, O. V. Sobolev, P. V. Afonine, P. D. Adams, Automated map sharpening by maximization of detail and connectivity. *Acta Crystallogr. D Struct. Biol.* **74**, 545–559 (2018).

42. N. W. Moriarty, R. W. Grosse-Kunstleve, P. D. Adams, Electronic ligand builder and optimization Workbench(eLBOW): A tool for ligand coordinate and restraint generation. *Acta Crystallogr. D Biol. Crystallogr.* **65**, 1074–1080 (2009).

43. D. Liebschner, P. V. Afonine, M. L. Baker, G. Bunkóczi, V. B. Chen, T. I. Croll, B. Hintze, L. W. Hung, S. Jain, A. J. McCoy, N. W. Moriarty, R. D. Oeffner, B. K. Poon, M. G. Prisant, R. J. Read, J. S. Richardson, D. C. Richardson, M. D. Sammito, O. V. Sobolev, D. H. Stockwell, T. C. Terwilliger, A. G. Urzhumtsev, L. L. Videau, C. J. Williams, P. D. Adams, Macromolecular structure determination using X-rays, neutrons and electrons: Recent developments in Phenix. *Acta Crystallogr. D Struct. Biol.* **75**, 861–877 (2019).

44. V. B. Chen, W. B. Arendall III, J. J. Headd, D. A. Keedy, R. M. Immormino, G. J. Kapral, L. W. Murray, J. S. Richardson, D. C. Richardson, MolProbity: All-atom structure validation for macromolecular crystallography. *Acta Crystallogr. D Biol. Crystallogr.* **66**, 12–21 (2010).

45. B. A. Barad, N. Echols, R. Y.R. Wang, Y. Cheng, F. DiMaio, P. D. Adams, J. S. Fraser, EMRinger: Side chain-directed model and map validation for 3D cryo-electron microscopy. *Nat. Methods* **12**, 943–946 (2015).

46. E. Krieger, G. Vriend, YASARA View—molecular graphics for all devices—from smartphones to workstations. *Bioinformatics* **30**, 2981–2982 (2014).

47. Y. J. Park, A. C. Walls, Z. Wang, M. M. Sauer, W. Li, M. A. Tortorici, B. J. Bosch, F. DiMaio, D. Veesler, Structures of MERS-CoV spike glycoprotein in complex with sialoside attachment receptors. *Nat. Struct. Mol. Biol.* **26**, 1151–1157 (2019).

48. Y. Chen, K. R. Rajashankar, Y. Yang, S. S. Agnihothram, C. Liu, Y.L. Lin, R. S. Baric, F. Li, Crystal structure of the receptor-binding domain from newly emerged Middle East respiratory syndrome coronavirus. *J. Virol.* **87**, 10777–10783 (2013).

49. Y. Watanabe, J. D. Allen, D. Wrapp, J. S. McLellan, M. Crispin, Site-specific glycan analysis of the SARS-CoV-2 spike. *Science* **369**, 330–333 (2020).

50. E. Krieger, G. Vriend, New ways to boost molecular dynamics simulations. *J. Comput. Chem.* **36**, 996–1007 (2015).

51. A. C. Pan, H. Xu, T. Palpant, D. E. Shaw, Quantitative characterization of the binding and unbinding of millimolar drug fragments with molecular dynamics simulations. *J. Chem. Theory Comput.* **13**, 3372–3377 (2017).

52. W. Humphrey, A. Dalke, K. Schulten, VMD: Visual molecular dynamics. *J. Mol. Graph.* **14**, 33–38 (1996).

53. T. T. N. Thao, F. Labroussaa, N. Ebert, P. V'kovski, H. Stalder, J. Portmann, J. Kelly, S. Steiner, M. Holwerda, A. Kratzel, M. Gultom, K. Schmied, L. Laloli, L. Hüsser, M. Wider, S. Pfaender, D. Hirt, V. Cippà, S. Crespo-Pomar, S. Schröder, D. Muth, D. Niemeyer, V. M. Corman, M. A. Müller, C. Drosten, R. Dijkman, J. Jores, V. Thiel, Rapid reconstruction of SARS-CoV-2 using a synthetic genomics platform. *Nature* **582**, 561–565 (2020).

54. M. Laue, A. Kauter, T. Hoffmann, L. Möller, J. Michel, A. Nitsche, Morphometry of SARS-CoV and SARS-CoV-2 particles in ultrathin plastic sections of infected Vero cell cultures. *Sci. Rep.* **11**, 3515 (2021).

55. J. R. Kremer, D. N. Mastrnarde, J. R. McIntosh, Computer visualization of three-dimensional image data using IMOD. *J. Struct. Biol.* **116**, 71–76 (1996).

56. M. G. de Carvalho, A. L. McCormack, E. Olson, F. Ghomashchi, M. H. Gelb, J. R. Yates III, C. C. Leslie, Identification of phosphorylation sites of human 85-kDa cytosolic phospholipase A2 expressed in insect cells and present in human monocytes. *J. Biol. Chem.* **271**, 6987–6997 (1996).

57. F. Ghomashchi, V. Brglez, C. Payré, L. Jeammet, S. Bezzine, M. H. Gelb, G. Lambeau, Preparation of the full set of recombinant mouse- and human-secreted phospholipases A2. *Methods Enzymol.* **583**, 35–69 (2017).

58. H. Ashkenazy, S. Abadi, E. Martz, O. Chay, I. Mayrose, T. Pupko, N. Ben-Tal, ConSurf 2016: An improved methodology to estimate and visualize evolutionary conservation in macromolecules. *Nucleic Acids Res.* **44**, W344–W350 (2016).

59. A. G. Wrobel, D. J. Benton, P. Xu, C. Roustan, S. R. Martin, P. B. Rosenthal, J. J. Skehel, S. J. Gamblin, SARS-CoV-2 and bat RaTG13 spike glycoprotein structures inform on virus evolution and furin-cleavage effects. *Nat. Struct. Mol. Biol.* **27**, 763–767 (2020).

60. J. Huo, H. Mikolajek, A. le Bas, J. J. Clark, P. Sharma, A. Kipar, J. Dormon, C. Norman, M. Weckener, D. K. Clare, P. J. Harrison, J. A. Tree, K. R. Buttigieg, F. J. Salguero, R. Watson, D. Knott, O. Carnell, D. Ngabo, M. J. Elmore, S. Fotheringham, A. Harding, L. Moynié, P. N. Ward, M. Dumoux, T. Prince, Y. Hall, J. A. Hiscox, A. Owen, W. James, M. W. Carroll, J. P. Stewart, J. H. Naismith, R. J. Owens, A potent SARS-CoV-2 neutralising nanobody shows therapeutic efficacy in the Syrian golden hamster model of COVID-19. *Nat. Commun.* **12**, 5469 (2021).
